# Proton pump inhibitors display anti‐tumour potential in glioma

**DOI:** 10.1111/cpr.13321

**Published:** 2022-08-12

**Authors:** Bihan Li, Ying Liu, Shilong Sun

**Affiliations:** ^1^ Department of Toxicology School of Public Health, Jilin University Changchun Jilin 130021 China; ^2^ NHC Key Laboratory of Radiobiology School of Public Health, Jilin University Changchun Jilin 130021 China

## Abstract

**Objectives:**

Glioma is one of the most aggressive brain tumours with poor overall survival despite advanced technology in surgical resection, chemotherapy and radiation. Progression and recurrence are the hinge causes of low survival. Our aim is to explain the concrete mechanism in the proliferation and progression of tumours based on tumour microenvironment (TME). The main purpose is to illustrate the mechanism of proton pump inhibitors (PPIs) in affecting acidity, hypoxia, oxidative stress, inflammatory response and autophagy based on the TME to induce apoptosis and enhance the sensitivity of chemoradiotherapy.

**Findings:**

TME is the main medium for tumour growth and progression. Acidity, hypoxia, inflammatory response, autophagy, angiogenesis and so on are the main causes of tumour progress. PPIs, as a common clinical drug to inhibit gastric acid secretion, have the advantages of fast onset, long action time and small adverse reactions. Nowadays, several kinds of literature highlight the potential of PPIs in inhibiting tumour progression. However, long‐term use of PPIs alone also has obvious side effects. Therefore, till now, how to apply PPIs to promote the effect of radio‐chemotherapy and find the concrete dose and concentration of combined use are novel challenges.

**Conclusions:**

PPIs display the potential in enhancing the sensitivity of chemoradiotherapy to defend against glioma based on TME. In the clinic, it is also necessary to explore specific concentrations and dosages in synthetic applications.

## Graphical abstract

Pump proton inhibitors (PPIs) produce a marked effect on the pump proton on stomach. However, nowadays, PPIs have been discovered the potential in anti‐tumour. Many kinds of cytokines and signal pathways in tumour microenvironment play an essential role in fight tumour. Oxidative stress, inflammation and autophagy are also main factors inducing proliferation and metastasis of tumour. PPIs have the ability in anti‐tumour through acting on oxidative stress, inflammation and autophagy based on cytokines and signal pathways to induce tumour cells damage. Anti‐cancer is a long process. However, using PPIs alone for a long time will have strong side effects. Therefore, at present, using PPIs to sensitize radiotherapy and chemotherapy is a valuable way. Most studies have shown the effect of PPIs on gastric cancer and lung cancer, but there are few studies on glioma. This research analyzes the concrete mechanism that PPIs can be used to treat glioma, so as to provide basis for clinical practice in the future.

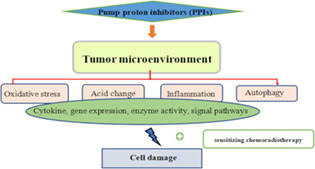

## 1 BACKGROUND

*Glioma* is one of the most aggressive brain tumours. Histologically, the World Health Organization (WHO) classified the tumours of the central nervous system (CNS) into astrocytomas, oligodendrogliomas and ependymoma. In addition, gliomas are classified into four grades according to the degree of malignancy. Types I and II are low‐grade gliomas and types III and IV are high‐grade gliomas.^1^, ^2^, ^3^ The clinical cure rate and 5‐year survival rate of patients are all very low. The prognosis is very dismal and the average survival period is only about 1 year. One of the main reasons for the poor prognosis of patients is that glioma is prone to drug resistance to chemoradiation therapy resistance.^4^, ^5^ Therefore, it is urgent to explore new strategies for glioma treatment.

*Pump proton inhibitors (PPIs)* are a drug of choice for inhibiting gastric acid secretion. In clinical, they are the first‐line option to treat peptic ulcers, gastroesophageal reflux disease, zoai syndrome and upper gastrointestinal bleeding.^6^, ^7^ It has become the first‐line drug for abnormal gastric acid secretion and related diseases combined with amoxicillin, clarithromycin and other drugs to treat *Helicobacter pylori* infection.^8^, ^9^ PPIs have the advantages of fast onset, strong acid inhibition, long action time, low blood drug concentration and low adverse reactions.^10^ The first generation includes omeprazole, lansoprazole and pantoprazole; the second generation includes iprazole, rabeprazole and esomeprazole. Compared with the first‐generation PPIs, in terms of drug properties, the second‐generation PPIs have the advantages of higher bioavailability, less affected by food and anti‐acid drugs, slow plasma clearance, less first‐pass effect after oral administration, higher stability, less adverse reactions and longer half‐life. In clinical, the second‐generation PPIs have better effects in inhibiting gastric acid secretion and *H. pylori*. Besides, they are more effective for relieving pain and the treatment of ulcers, especially, the coalescence of duodenal bulb ulcers. While combined with other medicines, the second‐generation PPIs revealed higher security and effectiveness.^11^, ^12^, ^13^

Progression and invasion are the main causes of death in malignant tumour patients.^14^ The tumour microenvironment (TME) is a multi‐complex environment regulated by various cytokines, transcription factors and pathways.^15^ One major cause of the progression and invasion is set off by the intimate relationship of glioma cells with the microenvironment. Recent therapeutic anti‐tumour approaches focus on such critical components of TME.^16^, ^17^

Thus, we searched Pubmed and Cnki conducting for this review. Our aim is to explain the specific relationship between TME and the progression of glioma. PPIs, the main force for treating the stomach, were found the proficiency to promote the sensitivity of glioma to chemoradiotherapy. Thus, associating with the mechanism of PPIs, another crucial purpose is to make clear the mechanism of PPIs in hypersensitization to inhibit proliferation and progression of glioma based on TME.^18^

## 2 EPIDEMIOLOGY OF GLIOMA

Glioma is the most common and malignant primary brain tumour in the CNS caused by canceration of glial cells in the brain and spinal cord. Up to the present, glioma is the most invasive and incurable cancer.^19^, ^20^ In the worldwide, about 100,000 cases of diffuse glioma are recorded every year. The annual incidence rate is about 3–8 cases per 10,000 persons.^21^ However, the incidence of glioma is increasing gradually. The peak incidence of primary glioma is between the ages of 55 and 60 years and the incidence rate is higher in men than women. Secondary GBMs tend to affect younger individuals about age 40 years old.^22^, ^23^ The main median survival is 15 months under treatment and 5 months without treatment, respectively.^24^, ^25^ However, the patients affected by low‐grade gliomas may survive for more than 20 years.^26^

Up to date, the mainstay of treatment methods contains surgical excision, chemotherapy and radiotherapy.^24^ According to the fifth edition of the WHO Classification of Tumours of the CNS (WHO CNS5), the standard of care and prognosis of glioma, circumscribed gliomas are usually benign and recommended for early complete resection, associated with chemotherapy if necessary. Diffuse gliomas and other high‐grade gliomas according to their molecule subtype are slightly intractable, with the necessity of chemotherapy. However, for glioblastoma, feasible resection followed by radiotherapy and temozolomide chemotherapy is contained in the current standard of care.^27^, ^28^

Glioma stem cells with the characteristics of stem cells and heterogeneous resident nerve cell spheres can promote the recurrence and progression of glioma.^29^ In terms of progression, there are two main methods of recurrence after surgical resection. The first is to convert the apoptosis‐related factor ligand (FasL) in astrocytes into the paracrine death signal pathway of cancer cells, or effect by inhibiting the axon Pathfinder L1 cell adhesion molecule (L1CAM) to promote the growth of glioma. Serine protease inhibitors (serpins) in glioma progression can inhibit the production of fibrinolytic enzymes and ensure the survival of cancer cells by protecting cancer cells from the biological process of death and promoting the growth of vessels.^30^

Moreover, with the extension of treatment time, chemoresistance occurs frequently. Also, it is inevitable for normal tissues adjacent to cancer to be damaged.^31^ Due to the resistance to chemoradiotherapy and the aggravation of glioma, local recurrence and distant progression are prone to occur.^32^ Moreover, because the glioma is prone to pass across the blood–brain barrier (BBB) adhering to the surface of capillaries and growing around capillaries, the delivery and penetration of therapeutic drugs passed into the brain are restricted resulting in the weakened therapeutic effects.^33^

As a result, the clinical therapeutic effect of glioma is deficient. Nowadays, it is an urgent problem to explore novel drugs with better curative effects to overcome the resistance and the physiological barriers.

## 3 THE CLINICAL APPLICATION OF PPIS

PPIs are kinds of stomach medicine with minimal side effects, generally.^34^ Clinically, they are used to remedy peptic ulcers, gastroesophageal reflux disease, zoai syndrome and upper gastrointestinal bleeding having become the first‐line drugs for abnormal gastric acid secretion and related diseases.^35^, ^36^, ^37^, ^38^ However, different PPIs have different clinical application tactics and blood–brain barrier penetration (Table 1).^39^, ^40^, ^41^, ^42^

**TABLE 1:** Molecular weight, dose in clinic, blood–brain barrier (BBB) permeability of pump proton inhibitors (PPIs)

PPIs	Omeprazole	Lansoprazole	Pantoprazole	Rabeprazole	Esomeprazole
Molecular weight	345.416	369.362	383.370	359.44	367.398
Dose in clinic	20 mg/day	15–30 mg/day	40 mg/day	10–20 mg/day	0–40 mg/day
BBB permeability	Omeprazole was able to penetrate the BBB and the time to reach the maximum concentrations is about 60 min in brain. Lansoprazole could penetrate the BBB and is similar with omeprazole. The time to reach the maximum concentrations is about 40–60 min in brain. Pantoprazole is under trail. Rabeprazole is under trail. Esomeprazole is able to cross the BBB. The peak serum concentration is reached 90–180 min after oral administration.				

PPIs are mostly derivatives of benzimidazole compounds.^43^ They are mostly weakly basic drugs with low original drug activity. After being absorbed into the blood, it is transported to the gastric mucosal parietal cells and finally reaches the acidic cavity of the secretory tube where the pH < 4.^44^ The technical drug is easy to be ionized and positively charged in this environment to play a preferable role.^45^ Due to the low membrane penetration, the drug is continuously aggregated and converted into the form of bioactive hyposulfonic acid and hyposulfonamide under the catalysis of acid, and then mixed with the sulfhydryl (sh) of vacuolar ATPase (V‐ATPase) dehydrating and coupling to produce an irreversible covalent disulfide bond to inhibit the H^+^ transport mechanism of the enzyme and acid secretion.^46^

PPIs are also H^+^‐K^+^ ATPase inhibitors which may act on the acid‐secreting tubules on parietal cells. The acid‐secreting tubules secrete acid in the way of H^+^ and K^+^ exchanging through H^+^‐K^+^ ATPase on the membrane to pump H^+^ out of the cells. PPIs cannot transfer the hydrogen of parietal cells to the gastric cavity and inhibit the formation of gastric acid.^44^, ^47^ Besides, PPIs bind to cysteine residues nearby the nucleotide‐binding domain of subunit A covalently resulting in the inactivation of V‐ATPase to inhibit acid content secreted.^48^ Only as new pump molecules are synthesized and inserted into the cell membrane can gastric acid secretion restart.^49^ Besides the application in gastrosia, PPIs has the potential in curing tumour such as epithelial ovarian and breast cancer nowadays.^50^, ^51^, ^52^, ^53^ Till now, the most used PPIs are to treat late‐stage tumours. Such as omeprazole could inhibit the progression of early colorectal adenoma to colorectal cancer and distal metastasis of advanced breast cancer.^54^, ^55^ However, there has been a study reporting PPIs administration could decrease the occurrence of the early‐stage gastric cancer induced by ulcerative differentiation.^54^ In addition to being used alone, PPIs are often used in combination with conventional chemotherapy drugs for cancer.

Omeprazole has the ability to inhibit V‐ATPase to change the acidic microenvironment and enhance the sensitivity of drug‐resistant cells to taxol. Besides, omeprazole combined with paclitaxel can significantly reduce the tumour volume in animal models with epithelial ovarian cancer.^50^ V‐ATPase is overexpressed in epithelial ovarian cancer compared with normal ovarian epithelial cells. YAP (Yes‐associated protein) is one of the major transcription activation factors playing an important role in regulating cell proliferation and organ development. YAP is overexpressed and is connected with the progression and multi‐drug resistance of the tumour. V‐ATPase D1 also known as ATP6V0D1, is the D subunit of the V0 domain. YAP is associated with V‐ATPase D1 to promote progression and MDA of the tumour. Esomeprazole could inhibit the expression of YAP and V‐ATPase D1, also the combination of YAP and V‐ATPase D1 to promote the sensitivity of tumour cells to conventional anticancer drugs such as PTX.^51^ In terms of breast cancer, as the effective chemotherapeutic drugs for breast cancer, raloxifene and doxorubicin combined with omeprazole, lansoprazole and pantoprazole, the viability of breast cancer cells is decreased and the apoptosis is enhanced obviously, compared with raloxifene and doxorubicin solely.^52^, ^53^

## 4 TUMOUR MICROENVIRONMENT IS THE MAIN MEDIUM FOR THE PROLIFERATION AND PROGRESSION OF TUMOUR

Progression is the most common phenomenon for patients with glioma which may lead to death. Progression occurs when tumour cells spread from the site of origin to another part of the brain. Progression is a multifactorial process that depends on metabolic changes, gene mutations and TME. Tumour cells, cancer stem cells, cancer‐associated fibroblasts (CAFs) and cytokines secreted by these cells, extracellular matrix proteins, blood vessels and various extracellular substances form a complex environment consisting of TME.^56^, ^57^ TME is a recognized significant key element affecting tumour occurrence, growth and progression considered as a unit so as to generate a dynamic communication with tumour cells.^58^, ^59^ Tumour cells can change and maintain their own survival and development conditions through autocrine and paracrine, so as to promote the growth and development of tumours.^60^ TME not only includes the structure, function and metabolism of tumour tissues but is also related to the internal (nuclear and cytoplasmic) and external environment of tumour cells.^61^ The main characteristics of the tumour microenvironment are acidification and hypoxia because of the imbalanced steady‐state.^62^ Therefore, tumours have intracellular alkaline pH and lower extracellular pH ranging from 7.2 to 7.4 and 6.5 to 7.1, respectively.^63^, ^64^ The acidic microenvironment strongly contributes to tumour progression by stimulating invasion and progression, inhibiting the immune surveillance of cancers and conferring chemoresistance.^65^ The acidic extracellular environment is conducive to tissue damage and the activation of destructive enzymes in the extracellular matrix (ECM) increasing the potential for tumour progression and acquiring cell phenotype of multidrug resistance (MDR).^66^ In order to maintain intracellular pH (pHi), the tumour cells have evolved powerful mechanisms to counteract cytoplasmic acidification and expel accumulated protons from cells, including Na^+^/H^+^ exchanger (NHE), carbonic anhydrase, monocarboxylic acid transporter (MCT) and proton pumps.^67^

Pump proton could keep the stability of H^+^ in and outside the microenvironment, and evade the change of intracellular acidity caused by bioenergy conversion.^68^ Proton pumps such as V‐ATPase, NHE and the carbonic anhydrase are upregulated in cancer cells.^69^ In tumours, V‐ATPase can pump protons out of cells, alkalize the intracellular environment and acidify the extracellular environment, so as to resist apoptosis and promote tumour aggressiveness.^70^ Increased activity of these pH regulators protects cells from changes in cell phenotype caused by pHi fluctuations by squeezing H^+^ in the extracellular space.^71^

The metastatic potential of tumour cells is affected by the relationship between tumour cells and ECM. Low pH activates and triggers the secretion of proteolytic enzymes including matrix metalloproteinase‐2 (MMP‐2), MMP‐9, a tissue serine protease, adamalysin‐related membrane protease, cysteine protease, cathepsin and gelatinase leading to the degradation and remodelling of ECM, so as to promote tumour invasion and progression.^72^

It is reported that the acidic environment after the metabolism of tumours is not conducive to normal cells and results in the immune escape of tumour cells.^73^ PPIs can directly change the quantity of T‐cell receptors (TCR) or major histocompatibility complex (MHC) so as to improve the recognition and elimination of tumour cells by T cells.^74^ Alkalizing the acidic environment of tumour cells can also improve the activity of other effector cells such as natural killer cells (NK) or natural killer T cells (NKT).^75^ Under the acidic environment, tumour cells can use the nutrition provided by autophagy of normal cells to meet the needs of tumour cell growth.^76^ PPIs can also inhibit autophagy resulting in the lack of nutritional supply for tumour cell proliferation and the death of the tumour.^77^ Compared with normal cells, the tumour cells could be more adaptable to the imbalanced pH environment.^78^ Because the V‐ATPase complex is mainly located at the edge of tumour cells which may induce the acidification of TME and furtherly promote the growth of cancer.^79^ In addition, the acidic TME provides extremely suitable living conditions for the proteolytic activity of cathepsins especially the lysosomal cathepsins, since the activity of most of the cathepsins could be enhanced in an acidic condition.^80^ And they could activate growth factors and proteases or degrade components of the extracellular matrix to promote progression. For instance, Cathepsin B (Cat B) can upregulate the function of MMPs which leads to the detachment of cells leading to the initiation of the cell migration.^81^

Akt (protein kinase B[PKB]) accumulates in mitochondria and phosphorylates pyruvate dehydrogenase kinase 1 (PDK1) on phospho‐NDRG1 (THR346) to inactivate the pyruvate dehydrogenase complex. And then this pathway turns tumour metabolism to glycolysis to antagonize apoptosis, inhibit oxidative stress and maintain the proliferation of tumour cells.^82^ Simultaneously, low pH could increase glycolysis to inspire progression. Glycolytic metabolites are the synthetic raw materials of biological macromolecules and the indispensable structural elements of new tumour cells. The increase of lactic acid produced by glycolysis will decompose and destroy ECM and promote progression.^83^

EMT is pivotal for wound healing, processes and its occurrence in cancer is known to aggravate invasion, migration and drug resistance in tumours. The occurrence of EMT causes the loss of epithelial characters of tumour cells and the transformation into mesenchymal‐like cells and thus enhancing tumour cell proliferation and motility and decreasing cell apoptosis.^84^ Transforming growth factor‐β (TGF‐β) has played a key role in deciding EMT.^85^ V‐ATPase may promote EMT induced by TGF‐β. What is more, low pH is more conducive for tumour cells to produce more TGF‐β and promote EMT which implies the growth of cancer.^86^

**FIGURE 1:**
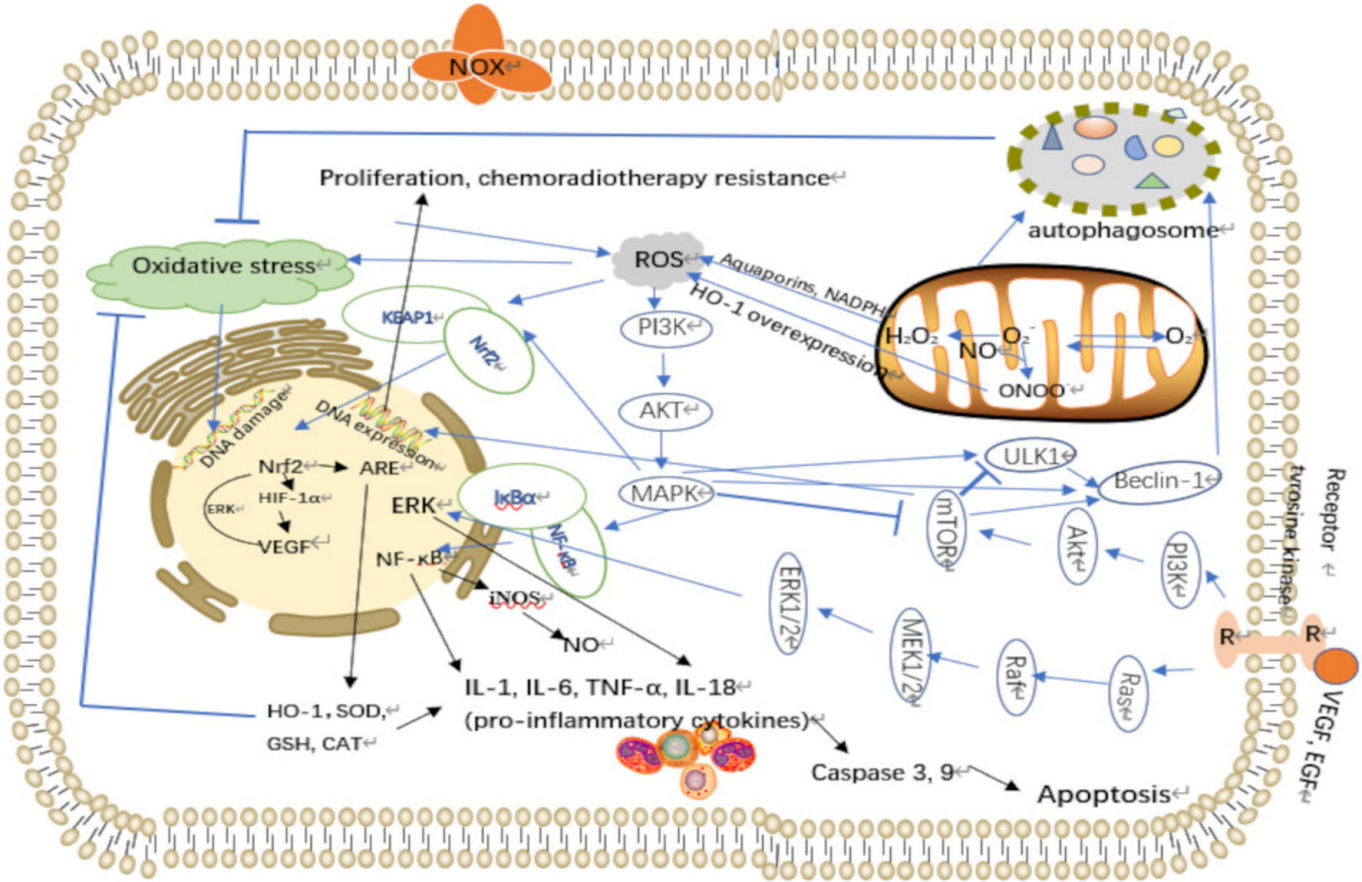
Schema depicting sources of ROS and antioxidant defences reported for glioma cells

*Hypoxia* is another major feature of TME associated with malignant progression, therapy resistance and poor prognosis of glioblastomas (Figure 1). Hypoxia is a pathophysiological condition that generally arises as a consequence of the rapid proliferation of cancer cells as they outgrow their blood supply, therefore, depleting the cells of nutrients and available oxygen. Hypoxia always happens in late‐stage tumours. Hypoxic tumours are found to be highly aggressive and resistant to chemoradiotherapy because hypoxic tumours require triple the normal radiation dose to achieve the desirable cell death effect as a normal irradiating dose which means that hypoxia is also a key factor in determining the growth of tumour‐induced by MDR.^87^, ^88^ Previous studies have revealed that oxygenation in glioma is 10 mmHg compared with normal brain tissue which is 40 mmHg and which may arise from radiation resistance.

Moreover, compared with normal cells, the expression of hypoxia‐inducible factor‐1α (HIF‐1α) and vascular endothelial growth factor (VEGF) in adjacent tissues was significantly higher.^89^, ^90^ Stably expressed HIF‐1α drives gene expression of two metabolic enzymes lactate dehydrogenase A (LDH‐A) and PDK1 which are vital in the conversion of pyruvate into lactate and inactivating pyruvate dehydrogenase (PDH) and subsequently prevent pyruvate oxidation in the mitochondria to promote glycolysis to improve the tolerance to hypoxia. This process could increase glucose to glycolysis while suppressing OXPHOS and mitochondrial respiration by decreasing the input into mitochondria to avoid apoptosis.^91^ It is the classical Warburg effect. The hydrogen ions removed from sugar will not be oxidized into the water through the respiratory chain, but will accumulate in large quantities in the cells, such as lactic acid.^92^ This altered glucose metabolism not only enables tumour cells to use glucose‐derived carbons for the synthesis of essential cellular ingredients but also rapidly provides ATP to fuel cellular activities. In addition, this metabolic shift contributes significantly to chemoradiotherapy resistance. Tumour cells can promote the Warburg effect, enhance acid secretion and increase extracellular pH (pHe) furtherly promoting the survival of tumour cells.^93^ Lactate accumulation also promotes cancer cell migration by facilitating the interaction of fibroblasts and escaping immune surveillance.^94^ At the same time, a large number of metabolites such as lactic acid and pyruvate can further improve the transcription and expression activity of HIF‐1α, and finally, form a positive feedback expression of HIF‐1α in a hypoxic environment (Figure 1).^95^ HIF‐1α degrades extracellular matrix and upregulates collagen expression genes promoting collagen fibre synthesis. HIF‐1α promotes the reconstruction of ECM under hypoxia based on proline 4‐hydroxylase A1 (P4HA1), P4HA2 and procollagen lysine oxoglutarate dioxygenase 2 (PLOD2) to increase directional migration.^96^ HIF‐1α regulates VEGF, TGF‐β and prospero homeobox‐1 (Prox‐1) to mediate the proliferation and progression of lymphatic endothelial cells and to disrupt the cellular barrier around existing blood vessels, pulling endothelial cells away to form new capillaries with fenestrations and fewer tight junctions. This results in an enhanced infiltration of cellular and plasma components into the brain, further inducing the reconstruction of TME, as well as maintaining the stem cell phenotype of tumour cells promoting progression and drug resistance.^97^, ^98^

In addition, nitric oxide synthases (NOS), widely expressed in gliomas, use l‐arginine to produce primary RNS type NO that interacts with O2^−^, generating ONOO^−^. NO is more effective in quenching superoxide and reacts with O_2_ to form other nitrogen oxides such as NO_2_, as a free radical, which in turn may react with NO to yield N_2_O_3_ reacting with biomolecules (lipids, proteins and DNA), potentially leading to cell death (Figure 1).^18^, ^99^ NO plays a role in the activation of NF‐κB which is a major transcription factor in glioma progression. IκB kinase‐independent NF‐κB activation may involve NO‐induced IκB nitration. RNS may disrupt the Keap1‐Nrf2 complex which can prolong the activation of Nrf2 and promote the antioxidant state inducing survival of tumour cells.^100^ RNS also inhibits wild‐type p53 through cysteine (Cys) oxidation and tyrosine (Tyr) nitration contributing to glioma genesis furtherly.^101^

**FIGURE 2:**
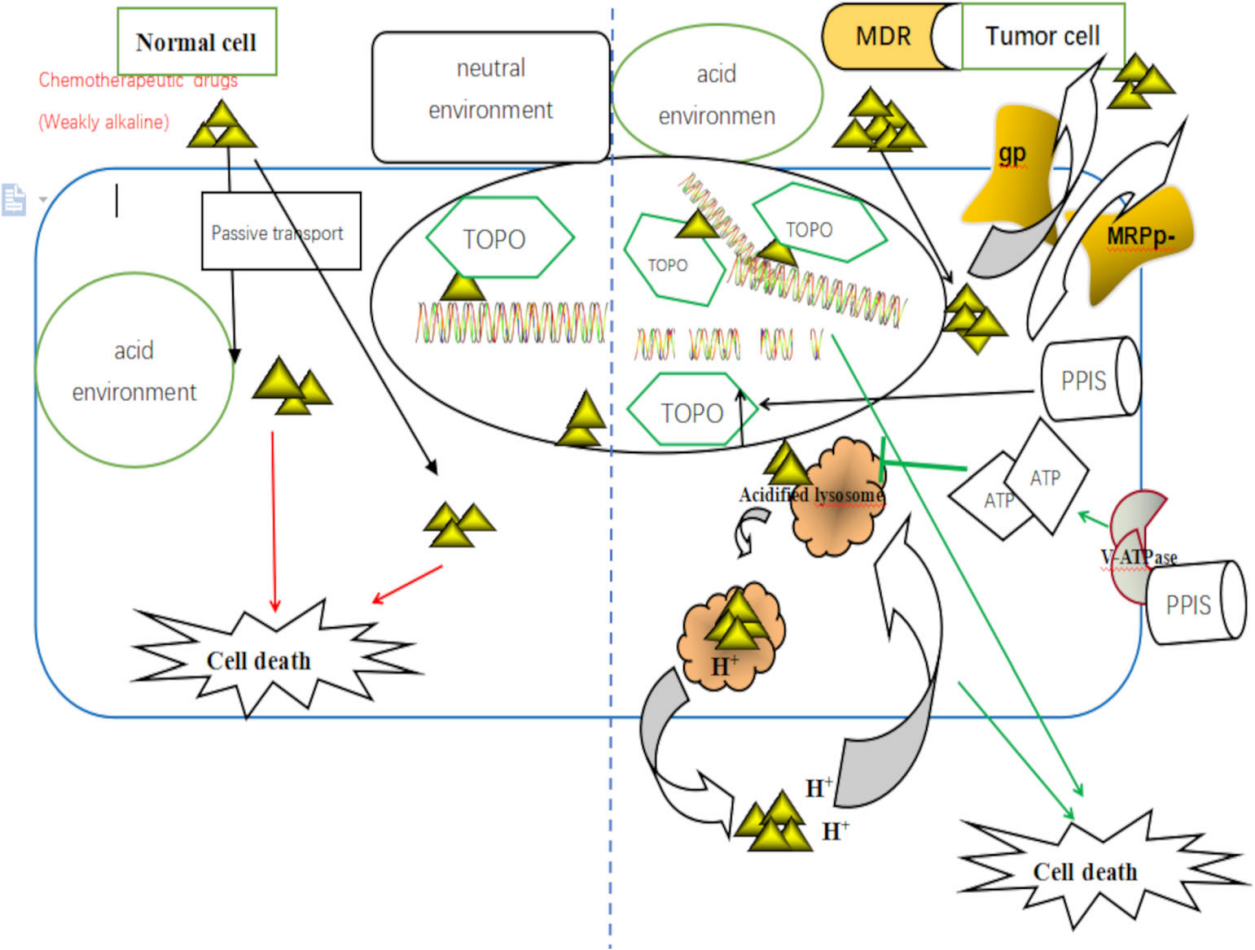
The mechanism of pump proton inhibitors (PPIs) for defending multidrug resistance

Another reason promoting progression and recurrence is MDR. A formidable obstacle for MDR is the blood‐tumour barrier (BTB) and blood–brain barrier (BBB), filtering barriers of capillaries. They exclude most compounds except highly lipidized small molecules of less than 400 daltons, rendering potentially powerful anti‐cancer drugs impotent for GBM treatment. Thus, breaking the BTB or BBB will significantly impact GBM treatment.^98^ Actually, the BBB is composed of non‐fenestrated brain endothelial cells (BECs) of the capillary wall, which is surrounded by pericytes, astrocytes, perivascular neurons, a basal membrane and an extracellular matrix, forming the highly organized neurovascular unit.^102^ Besides the nitrosourea compounds carmustine (BCNU) and lomustine (CCNU) as well as the platinum agents cisplatin and carboplatin, the most widely used chemotherapeutic drug is temozolomide (TMZ), an imidazotetrazine derivative of dacarbazine. TMZ could penetrate into the CNS and has 96%–100% bioavailability.^103^ However, the concentration of TMZ is far away efficient. TMZ is one of the substrates of P‐glycoprotein (P‐gp), an important efflux pump which locates on the apical membrane side of endothelial cells forming BBB and serves as a maintainer of the integrity and the polarity of BBB. P‐gp not only hinders brain entry of a large number of xenobiotics including potentially toxic substances and therapeutic agents but also transports the compounds that have crossed the BBB back into the circulation (Figure 2).^104^ Due to the overexpression of P‐gp at the BBB of glioma, only 20% of TMZ regarding a systemic dose is able to enter the cerebral parenchyma.^105^ In addition, BBB also accounts for the limited efficacy of other chemotherapeutic agents in GBM, such as etoposide, irinotecan, vincristine and cisplatin. Undeniably, BBB and BTB greatly contribute to the MDR.^106^ The mechanisms of MDR in tumour cells which is in the TME include four aspects: (1) The amplification or overexpression of transmembrane transporter gene leads to the high expression of encoded transmembrane transporter, so as to promote drug efflux and the change of drug subcellular distribution, resulting in the decrease of drug concentration in cells^107^ (2) the change of metabolic transformation, such as the change of some proteases in cells, result in the enhancement of cell detoxification (3) the change of drug action target, such as the decrease topoisomerase (TOPO) content or the change of property leads to the resistance to anti‐tumour drugs targeting TOPO (4) other mechanisms include the change of apoptosis‐related pathways, cell proliferation speed, the enhancement of damage repair and the change of pharmacokinetic factors.^108^ Several transmembrane transporter proteins may hinder drugs from reaching the target by reducing the concentration of drugs in tumour cells or changing the distribution of drugs in cells, such as drug efflux P‐glycoprotein (P‐gp), multidrug resistance protein (MRP). P‐gp is an efflux drug transporter associated with multidrug resistance gene‐1 (MDR‐1) expression in the cell membrane, which is responsible for expelling various drugs from tumour cells to form multidrug resistance.^109^ Weakly alkaline drugs can lead to capturing ions based on being protonated in an acidic extracellular environment, which hinders the effects of anti‐cancer chemotherapy drugs. In general, most chemotherapy drugs are weakly alkaline and the characteristics are prone to ionize in an acidic environment and with high polarity, so it is not suitable to pass through the cell membrane.^110^, ^111^ Once entering tumour cells, drugs are encapsulated in acidic organelles and the efficacy will be reduced or ineffective. Even several chemotherapy drugs induce the production of V‐ATPase in tumours maintaining the acid environment. Lysosomal vesicle or endosomes style structures in the acid environment can further expel drugs out of cells or eliminate drugs by activating relative secretory pathways. And then these structures can be further reused to enhance drug resistance limiting the drug's effects on its molecular targets (mainly DNA). Therefore, the concentration of chemotherapeutic drugs that can play a toxic and anti‐cancer role in cancer cells is still very low.^112^ In the hypoxia and glucose deficient tumour microenvironment, the expression of mRNA and protein of Topo is decreased and associated with decreased DNA breakage leading to the decreased cytotoxicity of chemotherapy drugs. At the same time, the content of complexes and chemotherapy drugs‐TOPO‐DNA is decreased resulting in MDR.^113^

The immune system's response to the tumour can impact the glioma's survival, proliferation and invasiveness.^114^ Tumour‐associated macrophages (TAMs) are formed by peripheral blood monocytes infiltrating into solid tumour tissues accounting for a large proportion of tumour stromal cells. TAMs are an important component of TME. It has been reported that *macrophages* are related to immunosuppression and immune escape.^115^ In the early stages, tumour cells release chemokines to attract macrophages and other inflammatory cells reaching the extracellular matrix. Then TAMs can penetrate the basement membrane so that tumour cells escape the bondage of the basement membrane and reach the surrounding normal tissue matrix.^116^ At the same time, TAMs and tumour cells can promote angiogenesis by releasing enzymes to generate angiogenesis, such as MMP2, MMP‐7, MMP‐9, MMP‐12 and cyclooxygenase‐2 (COX‐2) to improve the invasiveness and motility of cells.^117^, ^118^ Neovascularization can provide nutrition and oxygen for tumour growth and provide a path for tumour cell progression.^119^ TAMs also could release cytokines and growth factors directly to promote growth, such as VEGF, tumour necrosis factor‐α (TNF‐α) interleukin‐8 (IL‐8) and so on.^120^ Hypoxia in TME can further induce macrophages to produce HIF‐1α and promote angiogenesis.^121^

CAFs exist in the stroma of tumour cells which are associated with EMT and angiogenesis.^122^ Fibroblasts activate HIF‐1α and NF‐κB signalling pathways to stimulate oxidative stress, autophagy and glycolysis. These decomposition products create nutrient support for tumour growth.^123^ Besides, CAFs produce a variety of cytokines and extracellular matrix proteins including stromal cell‐derived factor 1 derived factor 1 (SDF1), hepatocyte growth factor (HGF), VEGF, platelet‐derived growth factor (PDGF) and TGF‐β to provide a basis for the progress and progression.^124^

*Mast cells (MC)* secret fibroblast growth factor 2 (FGF‐2), VEGF and TGF‐β to promote the progress and progression.^125^ A variety of cytokines produced by tumour cells can induce the proliferation of myeloid‐derived suppressor cells (MDSCs), such as COX‐2, IL‐6 and granulocyte‐macrophage colony‐stimulating factor (GM‐CSF) and VEGF can further enhance the immunosuppression of myeloid suppressor cells and enhance immune escape together.^126^, ^127^

This kind of neuro‐inflammatory TME can lead to the loss of BBB integrity.^128^ The consequences of a compromised BBB are deleteriously exposing the brain to potentially harmful concentrations of substances from the peripheral circulation, adversely affecting neuronal signalling and abnormal immune cell infiltration.^129^ All of these can lead to disruption of brain homeostasis.

*Autophagy* also displays a key role in the growth of glioma. Autophagy is an important catabolic process of substances in cells.^130^ It wraps the wrong proteins or damaged organelles through autophagy vesicles with double‐layer membrane structure, fuses with lysosomes and hydrolyzes with lysosomal acid hydrolases to produce biological molecules such as amino acids which are finally reused by cells to realize the circulation of substances in cells.^131^ In the early stage of tumour progression, autophagy will inhibit tumour progression. In the middle and late stages of tumour progression, autophagy will protect the tumour from stimulating and anoikis apoptosis so as to promote progression.^132^ Anoikis is a special form of programmed cell death induced by the loss of contact between cells and extracellular matrix and other cells. Usually, tumour cells gather together and closely adhere to the extracellular matrix to form a ‘home’ for self‐function and survival. When they break away from the cell adhesion matrix and lose the connection between cells, anoikis apoptosis will generate.^133^ Moreover, tumours will produce extensively damaged proteins, organelles and other harmful components after chemotherapy and radiotherapy. At this time, the activity of autophagy is improved to remove harmful substances in time and provide emergency and raw materials for DNA damage repairing which may result in a poor prognosis of tumour treatment.^134^

With the long‐term administration of chemistry, autophagy could gather the tumour cells to assemble into blocks promoting progression. Moreover, autophagy may induce the movement of damaged proteins, mitochondria and stressors including ROS to maintain the activation of advanced glioma.^135^ Chemotherapy could activate the autophagy genes like autophagy‐related protein 5 (Atg5), LC3 and others involved in autophagic pathways inducing progression.^136^, ^137^ PI3K‐Akt‐mTOR is a key signalling pathway that is related to autophagy.^137^ mTOR acts as the main regulator of autophagy containing two complexes called mammalian target of rapamycin complex 1 (mTORC1) and mammalian target of rapamycin complex 2 (mTORC2). MAPK inhibits mTORC1 activity, which leads to suppression of autophagy activating unc‐51‐like kinase 1 (ULK1). ULK1 induces the Beclin‐1 phosphorylation, which can result in autophagy.^138^, ^139^, ^140^ At the same time, autophagy is an adaptable response under the stimulation of the endoplasmic reticulum (ER). Under ER stress, Ca^2+^ is released into the cytoplasm and triggers autophagy by activating the MAPK‐TOR signal pathway. In addition, in the tumour microenvironment, cancer cells experience hypoxia resulting in the exposure of hydrophobic regions of misfolded/unfolded proteins and accumulation largely in the ER inducing the upregulation of unfolded protein response (UPR) and the expression of autophagy genes inducing invasion and progression. In addition to canonical UPR, proteotoxicity also stimulates the selective, autophagy‐dependent, removal of discrete ER domains loaded with misfolded proteins to further alleviate ER stress. These mechanisms can favour the progression and long‐term survival of advanced glioma cells.^141^, ^142^

It has been evidenced that autophagy could increase the expression of NF‐κB to activate MMP inducing invasion and progression.^143^ MMP plays a key role in the invasion process by degrading many elements of ECM, including collagens, fibronectin and laminin.^144^ It was reported that MMP is localized in vasculature cells and tumour cells of malignant astrocytomas.^145^ MMP inhibition significantly decreases invasion, migration and tumour progression in advanced glioma cells.^146^ All of the tumour cells would upregulate glycolysis to reduce the energy supplied by mitochondrial. Autophagy provides a source of energy in this process to promote the survival and progression of advanced glioma.^76^, ^147^

In terms of the pathological of gliomas, some mutations will indeed cause cells to continuously enter the cell cycle for mitosis, escape apoptosis, contact inhibition and immunosuppression and make cells continuously obtain energy so as to cause metabolic abnormalities and induce angiogenesis, hypoxia and necrosis of brain tumours, such as IDH mutation, H3K27M mutation, TERT mutation, MGMT mutation and so on. These mutations will activate the expression of various signal pathways and form the basis for the occurrence and development of glioma.^148^

### 4.1 PPIs can induce apoptosis by changing the acidic tumour microenvironment

**FIGURE 3:**
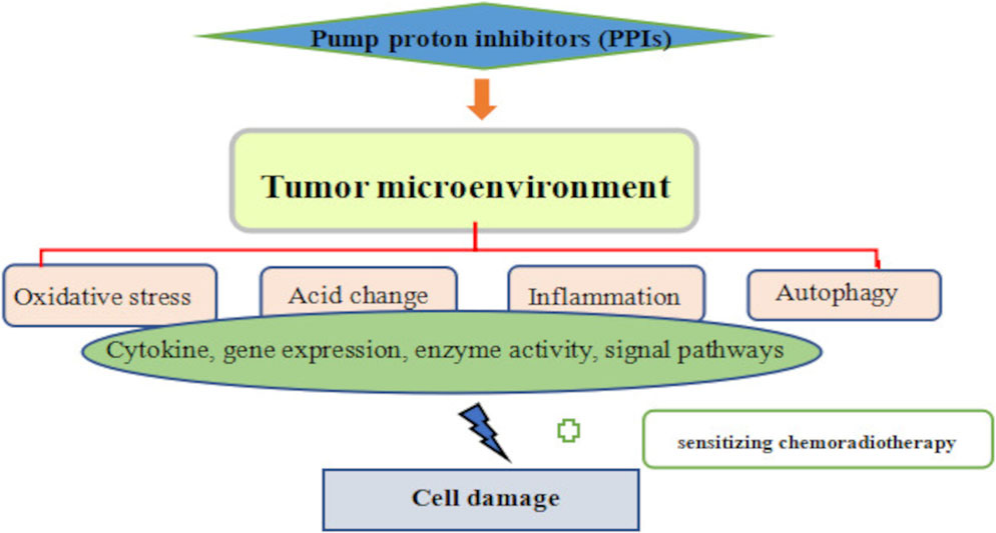
Mechanism of pump proton inhibitors (PPIs) inducing glioma cell damage

More and more studies have proved that the acidic environment outside the tumour cells plays an important role in the development, infiltration, dissemination and drug resistance of the tumour (Figure 3).^149^ PPIs inhibit the activity of V‐ATPase in the acidic environment outside the gastric cancer cells to hinder the proton transport, so as to change the pH gradient of gastric cancer cells and cause cell inactivation (Figure 3). The changed pH affects the structure and activity of almost every enzyme, then, these active enzymes affect various signals and pathways of cells.^150^ For example, V‐ATPase is involved in the Wnt/β‐Catenin signalling pathway which promotes tumour development and progression. Pantoprazole can inhibit V‐ATPase so that this pathway is blocked.^151^

The concrete mechanism of V‐ATPase is to maintain the balance of abnormal pH gradient inside and outside the cell.^152^ After being acidified outside the cell, V‐ATPase can activate proteolytic enzymes, including MMPs, tissue serine proteases and bone morphogenetic protease type 1 metalloproteinase (BMP‐1), to degrade ECM. V‐ATPase can also remove various toxic molecules, such as H^+^ and ROS.^153^, ^154^, ^155^ In addition, M‐ATPase and P‐ATPase play a similar role to V‐ATPase. These all exist on the cell membrane, mitochondrial membrane, lysosomal membrane, endosomal membrane and Golgi membrane, so as to maintain an acidic environment outside the cell membrane and inside the organelle membrane in gastric tumours.^156^ Omeprazole could cause the disorder of the lysosomal function of gastric tumour cells, further causing the activation of caspase‐3.^157^ The activated caspase‐3 cleaves the corresponding cytoplasmic and nuclear substrates, resulting in the apoptosis of the tumour finally.^158^ One research report that the rate of immune cell infiltration (M1 macrophages, neutrophils, CD103 cells and NK cells) is high, and the antitumor effectors (iNOS, INF‐γ, IL‐1α) are enhanced after using the inhibitor of V‐ATPase.^159^ At the same time, the number of cancer cell‐positive cells and the activity of caspase are all decreased.^155^ The role of PPIs is exactly similar to the inhibitor of V‐ATPase in suppressing the progression of the tumours. Likewise, esomeprazole, as an inhibitor of V‐ATPase, can reverse EMT by inhibiting IκB protein and furtherly inhibiting the expression of TGF‐β and thus countering tumour.^86^

The balance of acidity inside and outside cells can maintain the survival of normal cells. However, the acid–base imbalance is the main induced element of cell canceration. The main pH value is 7.2 and 7.4 in and outside the normal cells, respectively. However, the main pH value in and outside the tumour is 7.4 and 6.3, respectively.^85^ The tumour could balance this kind of microenvironment through the functional expression of several different molecular types of machinery. The migration and growth of solid tumours rely on the supplement of glucose.^160^ In the short term, the tumour cells could adapt to the decreation of glucose. But the long‐term reduction of glucose would increase the apoptosis of cancer cells. In order to combat the reduction of glucose, the change in the microenvironment may produce more lactic acid which may reduce the death of cells induced by a shortage of glucose.^161^ The increase of lactic acid could downregulate the G1/S transition process in the cell cycle and inhibit tumour cells in the G0 phase, so as to reduce the demand of tumour cells for energy and nutrients. At the transcriptional level, the G2/M checkpoint was also down‐regulated which could further aggregate tumour cells in the G0 phase.^162^ Lactic acid could also activate the autophagy process of cells to reuse intracellular substances to maintain cell survival.^163^, ^164^ At the same time, several studies illustrate that lactic acid also inhibits the apoptosis of tumour cells by maintaining the content of NADPH and high expression of anti‐apoptotic protein.^165^, ^166^ In a word, lactic acid can be the main reason leading to tumour cells escaping apoptosis. PPIs could harbour acid production to invert the proliferation induced by lactic acid.^167^

### 4.2 PPIs can induce apoptosis by changing the anoxic tumour microenvironment

Oxidative stress is a popular research factor to recover the tumour even the glioma. In general, the damage induced by oxidative stress is mainly due to the imbalance between the antioxidant defence system and the excessive formation of ROS.^168^ In the normal functional states, the production and removal of ROS are in a dynamic balance. However, if the production of ROS exceeds the removal as given an acute stimulation, then, the excessive free radicals will cause irreversible oxidation damage to the body.^169^ The main resources of ROS are mitochondria and cytoplasm.^170^, ^171^ Mitochondria mainly produce oxygen‐free radicals and non‐oxygen‐free radicals, such as O_2_^−^ and H_2_O_2._ ^172^ Of course, other cells are the secondary source of ROS in TME. Macrophages, neutrophils and tumours are the main generated resources of ROS. ROS generated by macrophages is considered as defending and phagocytosing tumours.^173^ Studies also show that activated monocytes contacting with tumours in TME could generate a high quantity of ROS producing more impact on DNA damage, metabolic activity, membrane structure and relative protein synthesis.^174^ A low level of ROS may activate cell proliferation and inhibit cell senescence and death. The high quantity of ROS may lead to the oxidative modifications of DNA, protein and lipid, finally, harming cells.^175^ A large amount of nitric oxide (NO) will produce peroxynitrite with a stronger oxidation effect with a superoxide anion. ONOO^−^ can irreversibly damage mitochondria by inhibiting the mitochondrial respiratory function and the activity of Na^+^/K^+^ ATPase and reduce the biological activity of NO participating in the enhancement of cell adhesion, proliferation, vasoconstriction accelerating the injury of arteriosclerosis.^18^ ROS oxidizes or peroxidases unsaturated fatty acids of which the function is to support the fluidity of the cell membrane damaging the permeability of cell membranes, such as membrane receptors, membrane proteins and ion channels.^176^ Furthermore, the nutrients absorbed by cells are reduced, such as vitamins, amino acids and inorganic salts, resulting in immunopathologic injury. ROS can break the N‐glycosidic bond on nucleotides and produce base‐free sites leading to the breaking of the main chain.^177^ It could also promote DNA to produce pyrimidine dimer.^178^ In addition, modification of base groups occurs, such as acetylation which further affects protein synthesis, cytoskeleton and DNA damage repair.^179^ When taking protein into consideration, ROS can break and crosslink protein or polypeptide chains resulting in protease inactivation and metabolic disorder.^180^ Besides, due to the high expression of SOD and catalase enzymes in glioma; there is an accelerated conversion of superoxide to hydrogen peroxide in tumour cells which makes astrocytes particularly sensitive to damage induced by ROS.^181^

PPIs have been used to modulate the pHi, disturb the mitochondrial membrane potential and produce excessive ROS leading to apoptosis (Figure 3).^182^ Several studies implied the ROS in the TME may promote more macrophages to inhibit the progression of tumour.^183^ In addition, PPIs have been applied to enhance the secretion of gastrin, and then enhance the secretion of insulin by islet cells, further inhibiting glycolysis and strengthening oxidative stress.^183^

Studies concluded that P‐ATPase plays a crucial role in defending against ROS.^184^ Glycolysislar V‐ATPase is overexpressed in tumour cells and metastatic cells to inhibit the generation of ROS in the tumour acidic microenvironment.^50^ PPIs could inhibit P‐ATPase and V‐ATPase to enhance the sensitivity of tumour cells to oxidative stress.^185^ Mild up regulation of mammalian target of rapamycin (mTOR) pathway activity can promote the production of cellular antioxidants, in turn, while over‐activation will promote the production of ROS. PPIs, such as esomeprazole, could reduce the over‐transduction of mTOR signals furtherly inhibiting the growth of cancer cells and prolonging lives.^186^

The activity of cytochrome c oxidase (COX) is associated with the survival period of glioma. The median survival time of patients with low tumour COX activity is short, while the median survival time of patients with high tumour COX activity is long.^187^ Because COX promotes the switch from glycolytic to OXPHOS metabolism. Increased COX activity in tumours has been associated with tumour progression after chemotherapy failure.^188^, ^189^ Recently, a variety of studies have considered the activity of COX as a prognostic indicator of glioma.^190^, ^191^ Pantoprazole blocked the level of COX inducing the increased depolarized mitochondria (Δψ m) and ROS levels.^149^ Moreover, PPIs, as the inhibitor of pump proton, have the potential of inhibiting the activity of mitochondrial electron transport complex, electron transport to reduce mitochondrial respiration and energy supplement so that promoting tumour cell death.^192^

Different PPIs own different mechanisms of inducing apoptosis. Bax and Bcl‐2 genes play an important role in apoptosis.^193^ Pantoprazole can inhibit the activation of the STAT3 pathway and downregulate its downstream cyclin D1 and Bcl‐2 accordingly so as to inhibit the proliferation of tumour cells and induce apoptosis.^194^ Omeprazole is different from pantoprazole. Experimental research shows omeprazole cannot induce the apoptosis of SGC‐7901 (from lymph node progression from a 56‐year‐old female patient with gastric adenocarcinoma) through the expression of Bax and Bcl‐2 related genes, but, through the decreased mitochondrial membrane potential after the action of ROS and caspase pathway. Apoptosis signalling pathways related to caspase activation include mitochondrial/cytochrome c (Cyt‐c) pathway, death receptor pathway and ER pathway. After the action of omeprazole, the expression of caspase‐3 in SGC‐7901 cells increased as time goes on indicating that omeprazole may activate caspase‐3 through the mitochondrial/cyt‐c pathway to induce apoptosis.^195^

### 4.3 PPIs can enhance oxidative stress based on NF‐κB, MAPK, Keap1/NRF/ARE, PI3K/Akt signal pathways

ROS affects metabolism mainly based on NF‐κB, mitogen‐activated protein kinases (MAPK), Keap1/NRF/ARE and PI3K/Akt signal pathways (Figure 1).^71^ At first, NF‐κB and MAPK pathways exert an essential implication in oxidative stress.^196^ Under no stimulations, the main components P50/65 and IκBα are active in the cytoplasm with the limitation of an inhibitor of κB (IκB) protein.^197^ While accepting a stimulation, IκB is phosphorylated by IκB kinase (IKK) and IκB detaches from NF‐κB, enabling NF‐κB dimers to enter the nucleus and express relative target genes actively, such as cytokines, COX‐2 and pro‐inflammatory proteins.^24^

The three main subfamilies of MAPKs are extracellular signal‐regulated kinase (ERK), c‐Jun N‐terminal kinase (JNK) and p38 MAPK which may modulate gene expression of nuclear Nrf2 and antioxidants enzymes mediated by ARE.^198^, ^199^ Moreover, ERK could be activated in cell growth and differentiation. P38 was involved in cell apoptosis and stress signal pathway.^200^, ^201^ PPIs selectively inhibited the phosphorylation of ERK and stimulated the phosphorylation of p38 in a time and dose‐dependent manner to sensitize apoptosis.^201^ When the above three enzymes are activated, they can further promote the phosphorylation of NF‐κB and stimulate the release of TNF‐α and IL‐6 which would promote the proliferation of tumours.^202^ Omeprazole has been proved to inhibit the activity of MAPK and NF‐κB and subside with the downregulation of TNF‐α, IL‐6 and SOD2 which may suppress the growth of tumours.^195^ MAPK pathways are downstream pathways of different growth factor receptors such as epidermal growth factor (EGF). EGF activates protein kinase C (PKC) thereby activating the Ras/Raf/MEK/ERK pathway. It has been reported that the Ras/Raf/MEK/ERK pathway is involved in mediating H_2_O_2_‐induced apoptosis in human glioma cells.^203^, ^204^, ^205^ It is well known that KRAS mutations contribute to cell proliferation and survival in numerous cancers, including glioma. One pathway through which mutant KRAS acts is an inflammatory pathway that involves the kinase IKK and activates the transcription factor NF‐κB. BRAF is a kinase that is downstream of KRAS and is predictive of poor prognosis and therapeutic resistance.^206^ However, there has been evidence that the inhibitor of V‐ATPase can inhibit the mutation of B‐Raf and the subsequent MAPK–ERK pathway to promote tumour apoptosis.^207^ IDH (Isocitrate dehydrogenase) mutations were mainly distributed in II, and III‐grade gliomas and secondary gliomas defined by WHO. It is closely related to methylguanine methyltransferase (MGMT) promoter methylation and TP53 mutation.^208^ IDH mutations can prevent cells from resisting γ Radiation, singlet oxygen, UVB radiation and other emergency damage, promoting the progression of glioma. In terms of mechanism, IDH mutations will affect the affinity of the enzyme, resulting in the decrease of the affinity between the enzyme and substrate. IDH mutants will compete with wild‐type IDH for substrate, and mutant IDH is more likely to combine with the substrate to form a dimer, leading to the accumulation of HIF‐ α and activation of downstream target genes including VEGF so as to promote tumour progression.^209^ IDH mutations are often used to predict early glioma. In classifying lower‐grade gliomas with IDH mutation, the V‐ATPase is overexpressed. It is also mentioned that the use of V‐ATPase inhibitors can inhibit the expression of the neurodevelopmental core transcription factor POU3F2, thereby reducing IDH mutations and inhibiting tumour proliferation and anti‐radiation.^210^ Therefore, PPIs have the possibility of inhibiting IDH mutation in glioma, thus reducing the occurrence of glioma. Till now, a clinical epidemiological survey has shown that esomeprazole can inhibit the methylation of tumour suppressor gene APC and increase the expression of APC in oesophageal cancer.^211^ With regard to glioma, some studies have shown that PPIs can inhibit MGMT promoter methylation, thereby increasing the sensitivity of glioma to radiotherapy and chemotherapy.^212^ Mutations in H3.3 often occur in glioma, resulting in decreased H3 histone methylation and increased H3 histone acetylation with subsequent activation of transcription promoting progression.^213^ Studies have shown that acetylation inhibitors can inhibit the pump protons and down‐regulate the effective components of the MAPK–ERK‐BRaf pathway so as to inhibit tumour proliferation. However, in terms of tumours resistant to acetylation inhibitors, the activity of related drug metabolic enzymes increases so as to promote anti‐tumour, after the administration of omeprazole, lansoprazole and pantoprazole. These further indicate that PPIs have the potential to inhibit tumour progression.^214^, ^215^, ^216^ One of the serious risks of glioma is epilepsy. Up to 75% of LGG patients have seizures during the course of the disease.^217^ Epilepsy is a transient interruption of normal electroencephalogram activity, which significantly affects the quality of life. The mass effect of glioma mainly refers to the excessive proliferation of brain white matter, which leads to the gradual increase of tumour volume.^218^ With the increased volume of tumours, it is easy to compress the surrounding brain tissue, and it is easy to cause the loss of stability and uncoordinated discharge of neurons during the growth of glioma. The two main reasons are IDH mutation and abnormal activation of the mTOR pathway.^219^ However, PPIs have the potential to suppress the IDH mutation and mTOR pathway. Besides, it has been proved by an experiment that the proportion of myelinated axons increased after omeprazole treatment. In vitro incubation with omeprazole significantly promoted the differentiation and maturation of oligodendrocyte precursor cells. In vivo, Omeprazole treatment (10 mg/kg) for 2 weeks significantly improved the motor coordination function of demyelinated mice.^220^ PPIs also could prolong action potential.^221^ Thus, PPIs may play a crucial role in preventing seizures.

Nrf2 is a key transcription factor regulating gene expressions of several antioxidant enzymes, such as NAD(P)H quinone acceptor oxidoreductase 1 (NQO1), SOD, catalase, glutathione peroxidase (GPX), glutathione reductase (GR) and haeme oxygenase‐1 (HO‐1), which play important roles in protecting cells against oxidative damage.^222^ Nrf2 also plays an important role in the tumour environment to promote the proliferation of glioma and protect glioma from anti‐tumour therapies.^223^ Tumour cells lose the heterozygosity of gene through Nrf2 or Keap1 mutation so that Nrf2 and Keap1 cannot be combined normally resulting in Nrf2 accumulation in tumour cells and then activating downstream genes increasing the level of detoxifying enzymes in tumour cells, promoting the formation and growth of tumour cells, enhance the resistance of tumour cells to radiotherapy and chemotherapy. Thus, blocking‐up Nrf2 could suppress glioma.^224^, ^225^ The microenvironment serves as the basis for indirect mechanisms of Nrf2 in the treatment of glioma. The mechanism of downregulating Nrf2 is mainly about two aspects: direct and indirect ways. Indirect mechanisms include three main aspects of the microenvironment: perivascular, hypoxic and immune microenvironment.^226^ Angiogenesis plays a key role in providing energy so as to activate the proliferation of glioma. Nrf2 was found to significantly increase microvessel density (MVD) and expression of small vessel marker CD31.^227^ Nrf2 could also regulate angiogenesis based on HIF‐1α and VEGFs.^228^ HIF‐1α is a downstream molecule of Nrf2 to regulate hypoxia. Activation of HIF‐1α could activate numerous perivascular compounds, such as angiopoietin, endothelin‐1, inducible nitric oxide synthase (iNOS), adrenomedullin and erythropoietin. Furtherly, in turn, VEGF can also activate Nrf2 according to activate ERK1/2 and induce the production of antioxidative enzymes.^229^, ^230^ The glioma is addicted to an anoxic environment. The overexpression of Nrf2 exerts antioxidant function and further promotes the expression of HIF‐1α and HO‐1(HO‐1 is a molecule to resists hypoxia) to inhibit the migration and invasion of tumours in a hypoxic microenvironment finally.^231^ HO‐1 also plays a key role in fighting against inflammation via ERK/Nrf2 signal cascade induced by oxidative stress.^232^

Glioma could evade immune surveillance to decrease the response between the tumour cells and immune surveillance cells. Nrf2/ARE pathway regulates tumour immune surveillance based on regulating the secretion of cytokines and the function of immune cells.^233^ Nrf2 regulates the secretion of a variety of cytokines, such as INF‐γ, IL‐5 and IL‐13.^234^ INF‐γ induces the production of cytokines affected by immunosuppression and growth factors, which is conducive to the growth and progress of tumour cells. Besides, it may downregulate Alpha‐fetoprotein (AFP) and melanoma antigen (MAGE) to promote antigen modulation of tumour cells so as to escape immune surveillance.^235^ At the same time, Nrf2 could induce the transformation of CD4 (+) T cells into the T helper cells 2 (Th2) to secret IL‐4 and IL‐10 in order to inhibit immune protection.^236^ The above indirect mechanisms all rely on vascular endothelial cells, fibroblasts and immune cells which are all existed in TME.^19^ Nrf2 may active proteasome protective genes such as Phase II detoxification enzyme gene, proteasome gene, ubiquitinase gene, antioxidant protein gene and multidirectional drug‐resistant protein 2 (MRP2) gene which have been proved to inactive external substances and detoxify and be associated with MDR.^237^ Besides, under low oxidative stress, overexpressed Nrf2 will reduce the sensitivity to cytotoxic chemotherapeutic drugs by promoting the detoxification of anti‐cancer drugs and enhancing the antioxidant capacity.^238^, ^239^ Based on the above mechanisms, PPIs have been proven to inhibit the activation of Nrf2 and downregulate the expression of genes or enzymes regulated by Nrf2 to suppress the growth of glioma, such as SOD, GPx, catalase (CAT), HO‐1 which may inhibit antioxidation.^240^

The acidic microenvironment can inhibit the production and function of CD4, CD25, factor Forkhead box P3 (FOXp3) and iT‐regs through the PI3K/Akt/mTOR signal pathway.^241^ IL‐10 produced by iT‐regs decreased the production of ROS in the vascular wall, the activity of NADPH and improved vascular endothelial dysfunction.^242^ However, it has been proven that omeprazole and pantoprazole have the ability to reduce the iT‐regs differentiation in an acidic microenvironment so as to suppress the glioma.^241^

### 4.4 PPIs can inhibit tumour progression by inhibiting tumour‐related inflammation

Enhanced inflammation is a risk factor for many cancers. Immunosuppression caused by inflammation is one of the main reasons for poor prognosis and the short survival of glioma patients.^238^ Macrophages are natural immune cells that can be found in most TMEs.^243^ Research reported that macrophages produce a small number of tumorigenic factors in vitro, and their ability to inhibit T‐cell activation and proliferation is reduced and associated with the decreased of Dew sugar receptor‐1 (CD206), IL‐10, TGF‐β, the expression of arginase‐1, matrix metalloproteinase and vascular endothelial growth factor after using sh‐V‐ATPase.^244^, ^245^ Being similar to the inhibitor of V‐ATPase and sh‐V‐ATPase, PPIs may play a similar role in inhibiting tumour progression.

In different microenvironments, there are different macrophage subtypes containing pro‐inflammatory and anti‐inflammatory. M1 subtype could promote macrophage phenotypes to inhibit tumour. M2 subtype could suppress macrophage phenotypes to promote tumour.^246^, ^247^ Research has proved that pantoprazole may enhance recruitment of TAM in TME showing augmented expression of CD11c, phagocytosis and macrophage morphology.^248^, ^249^ Phagocytosis and expression of CD11c are considered important signature markers of macrophages with M1 phenotype.^250^ Additionally, pantoprazole displays increased anti‐tumour activity with an augmented expression of anti‐tumour molecules, such as IL‐1, TNF‐α, IL‐2R and NO. Experiments show that pantoprazole could counter the tumour as exposed in vitro. Thus, PPIs may not directly enhance the anti‐tumour activity, but rely on the cytokines secreted by macrophages indirectly.^251^

Inflammatory mediators can change the local environment of tumours, and chronic inflammation can lead to DNA damage.^252^ Clinically, anti‐inflammatory drugs can reduce the incidence of cancer.^85^ Especially, PPIs have been studied to target TNF‐α monoclonal antibodies, anti‐sensory targeting Smad7, and non‐steroidal anti‐inflammatory drugs to prevent cancer based on inflammation (Figure 3).^253^ Some inflammatory mediators and chemokines are special inflammatory mediators. PPIs directly inhibit epithelial cells and neutrophils or the production of relative chemokines playing an anti‐inflammatory role through suppressing the secretion of INF‐α and proinflammatory mediators such as IL‐1 and translationally controlled tumour protein (TCTP) so as to inhibit cancer‐related symptoms and progress.^254^ Besides, STAT3 plays an important role in maintaining and promoting the tumorigenic inflammatory environment, the transformation and progression of malignant tumours based on NF‐κB and IL‐6/GP130/JAK pathways.^255^ There has been a research reporting that pantoprazole can inhibit the activity of the IL‐6/STAT3 pathway in gastric cancer cells.^192^

TNF‐α and IL‐6 are two kinds of cytokines that play an important role in the process of cancerous cachexia.^256^, ^257^ IL‐6‐like cytokines independently mediate the excessive lipolysis and metabolism in cancerous cachexia.^258^ TNF‐α downregulates the PLIN pathway by upregulating the MAPK pathway so as to promote lipolysis. Abnormal metabolism of fat has a lot to do with the progression of tumours.^259^ Several relative experiments showed that TNF‐α and IL‐6 declined apparently in the serum of cancer cells in mice after intragastric omeprazole which implied that PPIs inhibit the tumour progression caused by fat metabolism.^254^ Besides, PPIs may decrease the quality of IL‐4 directly and furtherly stimulate the production of TNF‐α.^260^ Omeprazole and pantoprazole have been already proved to decrease pro‐inflammatory factors such as TNF and IL‐6, and increase anti‐inflammatory factors such as IL‐10 implying the potential for anti‐tumour.^193^ As a result, PPIs may give play to anti‐inflammatory and cancer clearance. Furthermore, PPIs could transfer M2 macrophages to M1 macrophages to fight cancer.^261^ Also, PPIs have a direct effect on neutrophils, monocytes, endothelial cells and epithelial cells, so as to exert an anti‐inflammatory effect and inhibit INF‐α, secretion of proinflammatory mediators such as IL‐1 and TCTP.^262^ The anti‐cancer activity of PPIs is based on the fact that it can significantly reduce the production of acid‐active substances, IL‐6 and nitric oxide, especially TNF‐α, expression of inducible NOS and COX‐2. PPIs can act in an acidic environment by inhibiting inflammatory factors to further inhibit cancer.^263^

### 4.5 PPIs display an anti‐tumour effect through autophagy

V‐ATPase is the main proton pump that acidifies vesicles such as lysosomes. PPIs can disrupt the lysosomal localization of V‐ATPase leading to lysosomal dysfunction, thus contributing to the lysosomal storage disorders in order to counter tumours.^264^

The mammalian target of rapamycin (mTOR) is a highly conservative serine/threonine‐protein kinase that belongs to the PI3K‐related protein kinase family.^112^ MTOR can phosphorylate Atg13 preventing it from interacting with complexes containing Atg1 and Atg17, and inhibiting autophagy assembly so as to reduce radiation resistance.^265^ The activation of mTOR could induce aerobic glycolysis through upregulating Pyruvate kinase M2 (PKM2), as well as the activation of OXPHO.^266^ MTOR can also stimulate the metabolism of lipids, nucleotides and other components to promote tumour progression. Moreover, mTOR can activate HIF‐1α promoting the regeneration of blood vessels.^267^ MTORC2 can also directly promote tumorigenesis by activating Akt or Serum‐ and glucocorticoid‐inducible kinases (SGK) promoting the growth of tumours.^268^

Hypoxia plays a crucial role in regulating autophagy since the absence of oxygen leads to inhibition of mTORC1 and subsequently decreased inhibition of the unc‐51 like Ulk1 complex finally resulting in activation of autophagy.^269^ PPIs depolarize mitochondrial membrane potential by increasing the opening of mitochondrial permeability transition pore (MPTP), resulting in excessive production of ROS which further stimulates lipid peroxidation and reduces glutathione levels.^270^ This process could generate excessive ROS leading to lysosomal chamber instability which will affect mitochondrial membrane potential and mitochondrial energy supplement to suppress hypoxia and autophagy in advanced glioma promoting the anti‐glioma.^270^ In addition, more ROS will promote the release of pro‐apoptotic molecules into the cytoplasm and further promote autophagy.^271^ It has been reported that PPIs can induce autophagy by activating ERK and JNK and inhibit mTOR signalling by activating MAPK.^201^, ^272^ There is also evidence showing that PPIs mediate the strong upregulation of Beclin‐1 by activating NF‐κB which is responsible for the ROS‐induced autophagy making the foundation for anti‐tumour.^273^, ^274^

Besides, PPIs could moderate the acidic microenvironment to inhibit the activation of lysosomal cathepsins to suppress MMP increasing sensitivity to TMZ. Finally, the progression of the tumour was inhibited.^275^ PPIs may activate MAPK kinase, reduce blood glucose levels and block glycolysis induced by TNF‐α so as to invert the Warburg effect which could activate the autophagy promoting progression.^276^

## 5 PPIS SENSITIZE CHEMORADIOTHERAPY PROMOTING APOPTOSIS

Chemoradiotherapy resistance is an important element to early recurrence, treatment failure and dismal prognosis of glioblastoma.^277^ With growing amounts of evidence that chemoradiotherapy may induce the more normal cells to glioma leading to recurrence and progression.^278^, ^279^ Even if the concentration of drugs entering plasma and cerebrospinal fluid is enough to inhibit the progression of the tumour, the drug resistance and progression of glioma also happen from time to time.^280^ Thus, hoping to make use of PPIs and chemoradiotherapy together to play a good effect.

The radiation may lead to the mutation of mitochondrion respiratory chain complex I and II and DNA which will lead to more ROS in glioma.^281^ Excessive ROS could damage DNA containing mitochondrion DNA leading to respiratory chain dysfunction, even higher levels of free radicals in the cells, and the disbalance of O_2_^−^ in and outside the cells, and, further, block oxygen supplement.^282^ In addition, ionizing radiation (IR) will produce more free radicals which combine with oxygen and further lead to permanent DNA damage.^283^ Thus, radiotherapy can irreversibly damage DNA through reactive oxygen species. However, the tolerance of normal cells to radiation dose is limited, and the radiation dose cannot be increased continuously.^284^ Hypoxic tumour cells continue to exist after radiation and transform into a more invasive phenotype.^19^ PPIs such as esomeprazole could generate more ROS persistently to make up for the deficiency of oxidative stress caused by limited radiation.^285^ The inhibitory effect of many chemotherapeutic drugs depends on the oxygen partial pressure of the TME. In general, the sensitivity of chemotherapy was the strongest at the peak of oxygen partial pressure.^286^, ^287^ Because of the ability to enhance oxygen partial pressure through enhancing oxidative stress, PPIs could increase the sensitivity to chemotherapy. However, we need to explore the dose and concentration of different PPIs with the maximum oxygen partial pressure in TME.

The inverted pH gradient of cell membrane inside and outside tumour is an important mechanism leading to drug resistance.^288^ The acidic extracellular microenvironment makes a chemical and physical shelter for the anti‐tumour effect of weakly alkaline chemotherapeutic drugs. Once the chemotherapeutic drugs are protonated and alkalized, they cannot pass through the plasma membrane.^241^, ^272^ Chemotherapeutic drugs in tumour cells enter lysosomes, acidic organelles, and acidic vesicles through protonation and neutralization, and are further discharged out of cells.^289^ PPIs changing cell microenvironment can inhibit the intracellular P13K/Akt/mTOR signal pathway and bypass the TSCL/2‐Rheb signal pathway to inhibit its downstream mTOR molecules, and then inhibit the expression of HIF‐1α, MDR protein and P‐gp.^290^, ^291^ In glioma, MDR protein and P‐gp gene are overexpressed.^292^ P‐gp could combine with chemotherapeutic drugs to pump the substrate out of the cell by hydrolyzing ATP, so as to inhibit the effect of chemotherapeutic drugs. Being similar to P‐gp, overexpressed multidrug resistant‐associate protein‐1(MRP1) pumps the drug out of cells.^293^ Research implied that PPIs may inhibit the expression of MRP1 and P‐gp to reverse chemoresistance. The excessive PPIs could be cultured in vitro by the alkalizate microenvironment of cancer cells so that increasing chemosensitivity.^294^ PPIs can reverse the Warburg effect of tumours by inhibiting pyruvate dehydrogenase kinases and subsequently activating mitochondrial OXPHO or changing anaerobic digestion and ATP binding box (ABC) transcription factors, so as to increase the sensitivity of cancer cells to chemoradiotherapy inducing apoptosis.^86^, ^295^

PPIs can prevent the occurrence of EMT by interfering with the expression of the TGF‐β/Smad signal pathway and NF‐κB in the acidic microenvironment.^296^, ^297^, ^298^, ^299^ It also has been proved that rabeprazole administration induced cell death and reduced cell migration together with EMT by inhibiting Akt and Gsk‐3β phosphorylation, which in turn suppressed the EMT. In addition to molecular aberrations and hypoxia, pump protons may facilitate epithelial to mesenchymal transition by modulating the pH of the TME. We found rabeprazole could play a better role in the unbuffered acidic environment to inhibit EMT.^300^ What is more, pantoprazole inhibits breast cancer resistance protein (BCRP) and has a chemo‐sensitizing activity, thereby contributing to improved delivery of imatinib (a kind of drug for treating leukaemia) into the CNS.^299^ It has been proved that treatment of the cancer cells with the PPIs resulted in order of magnitude reduction in the half‐maximal inhibitory concentration (IC_50_) values.^301^ Esomeprazole enhances radiosensitivity in radiation‐resistant squamous cell carcinoma of the head and neck.^285^ PPIs could also increase chemotherapeutic drug uptake by the tumour cells.^302^ PPIs, as the small molecules, could pass through BBB and BTB (Table 1).

In the clinic, PPIs have been proven the ability to overcome the resistance of conventional chemotherapeutic drugs for breast cancer and radiation. The use of PPIs in 6754 patients suffering from breast cancer significantly not only improved the overall survival rate of these patients and reduced the disease recurrence rate but overcome the resistance of conventional chemotherapy drugs and radiation.^303^ Besides, based on clinical epidemiological comparative trials, PPIs can assist radiotherapy and chemotherapy to delay the overall survival of 206 patients with rectal cancer.^56^ What is more, Omeprazole not only improved the effect of radiotherapy and chemotherapy but also delayed the recurrence and complications of 125 rectal cancer patients.^304^ The combined use of PPIs in the treatment of laryngeal cancer can reduce the incidence of pharyngeal reflux and mucositis.^305^ Although there has been no relative research studying the synergistic effect of chemoradiotherapy and PPIs in glioma, based on the above‐mentioned relevant clinical epidemiological study about the administration of PPIs in other cancers and preclinical pharmacologic or toxicologic metabolism and mechanism analysis. Of course, improper dosage selection of PPIs and chemoradiotherapy will also lead to the aggravation of adverse reactions. A clinical study showed that the inappropriate dose of PPIs led to the further aggravation of radiation pneumonia after radiotherapy for lung cancer.^306^

In general, PPIs may be a potential valuable anti‐cancer drug to promote chemoradiotherapy sensitivity so as to block the growth of glioma.

## 6 THE ACUTE SIDE EFFECT OF PPIS AND THE NECESSITY OF COMBINING PPIS WITH CHEMORADIOTHERAPY

The basic side effects of PPIs contain nausea,^307^ diarrhoea or headache,^308^ and even more serious side effects such as subacute cutaneous lupus erythematosus,^309^ and interstitial nephritis and pneumonia.^310^ After a long period of the epidemiological follow‐up survey, patients with long‐term use of PPIs may get complications of suppurative liver abscess, pulmonary tuberculosis and inflammatory bowel disease.^311^ Inflammatory mediators can cause metabolic disorders as being with PPIs for a long time, such as IL‐1, IL‐6, TNF‐α, INF‐γ and prostaglandin E2 (PGE2) which may induce an increase in muscle decomposition and a decrease of muscle synthesis lead to the decrease of muscle mass and muscle strength, even furtherly subside with decreased physical function, polymyositis and rhabdomyolysis.^312^ Besides, mitochondrial would be impaired and intestinal microbial would change based on the proximal pH of the intestine increases.^313^ It has been proved that PPIs elevate the pH of the stomach and upper gut causing more bacteria, even pathogenic bacteria, to survive in the gastrointestinal tract and enter the gut gradually.^314^ Vitamin B12, vitamin C, vitamin D, magnesium, calcium, iron, β‐carotene and zinc will be reduced due to the decreased relative quality of food release and body absorption.^315^, ^316^, ^317^, ^318^, ^319^ Suppressing magnesium may inhibit the absorption of Vitamin D implying the release of adipocytokine which is involved in the regulation of glucose levels and fatty acid breakdown inducing insulin resistance so that the appearance of obesity and contribute to an increase of the inflammatory state.^315^ Based on a reported real case, PPIs may be associated with several mental diseases.^320^ Thus, it is urgent to design specific and efficient combined medication or united anti‐glioma therapies to treat glioma.

## 7 CONCLUSIONS AND FUTURE DIRECTIONS

The tumour microenvironment is the main medium for proliferation, progression and multidrug resistance of tumours. Acidity and hypoxia, as well as the relative inflammatory response and autophagy, are the main influencing factors for tumour progression and progression in TME. PPIs could promote apoptosis and sensitize chemoradiotherapy through altering the release of inflammatory factors and cytokines of inflammatory cells, autophagy and promoting oxidative stress based on NF‐κB, MAPK, Keap1/NRF/ARE, PI3K/Akt signal pathways in TME. PPIs could be considered as an adjuvant treatment strategy to be combined with medication to fight glioma. In order to better predict the therapeutic effect, COX can be considered a prognostic indicator.

In terms of the practical application scope of PPIs nowadays, many kinds of PPIs have been used for different cancer, such as liver, and breast cancer. Nowadays, there is little known about its possible glioma protective effects and relatively few studies on practical application in glioma. Therefore, it is necessary to put PPIs into practice to determine the real advantages and disadvantages of PPIs.

## AUTHOR CONTRIBUTIONS

Bihan Li collected, read relevant literature and wrote the article. Ying Liu collected relative literatures and guided this article. Shilong Sun collected relative literatures and guided the mentality and this article. [Correction added on 22 March 2023, after first online publication: Author Contributions have been amended.].

## CONFLICT OF INTEREST

The authors declare no conflicts of interest.

## Data Availability

Data sharing is not applicable to this article as no new data were created or analyzed in this study.

## References

[1] Zhou YS , Wang W , Chen N , Wang LC , Huang JB . Research progress of anti‐glioma chemotherapeutic drugs (review). Oncol Rep. 2022;47(5):101. doi:10.3892/or.2022.8312 35362540 PMC8990335

[2] Hua T , Zhuo Z , Duan Y , et al. Prediction of H3 K27M‐mutant in midline gliomas by magnetic resonance imaging: a systematic review and meta‐analysis. Neuroradiology. 2022;64:1311‐1319. doi:10.1007/s00234-022-02947-4 35416485

[3] Grossen A , Smith K , Coulibaly N , et al. Physical forces in glioblastoma migration: a systematic review. Int J Mol Sci. 2022;23(7):4055. doi:10.3390/ijms23074055 35409420 PMC9000211

[4] Sisakht AK , Malekan M , Ghobadinezhad F , et al. Cellular conversations in glioblastoma progression, diagnosis and treatment. Cell Mol Neurobiol. 2022;101:31‐33. doi:10.1007/s10571-022-01212-9 PMC11415179 35411434

[5] Ribatti D . The chick embryo chorioallantoic membrane as an experimental model to study in vivo angiogenesis in glioblastoma multiforme. Brain Res Bull. 2022;182:26‐29. doi:10.1016/j.brainresbull.2022.02.005 35143927

[6] Tyron JM , Eliasen A , Dalhoff K , Nørgaard MM . The efficacy and safety of proton pump inhibitors in infants with reflux. Ugeskr Laeger. 2022;184(15):V08210634. 35410647

[7] Clarke K , Adler N , Agrawal D , et al. Indications for the use of proton pump inhibitors for stress ulcer prophylaxis and peptic ulcer bleeding in hospitalized patients. Am J Med. 2022;135(3):313‐317. doi:10.1016/j.amjmed.2021.09.010 34655535

[8] Liou JM , Lee YC , Wu MS . Taiwan gastrointestinal disease and helicobacter consortium. Treatment of refractory *helicobacter pylori* infection‐tailored or empirical therapy. Gut Liver. 2022;16(1):8‐18. doi:10.5009/gnl20330 33782215 PMC8761919

[9] Sue S , Maeda S . Is a potassium‐competitive acid blocker truly superior to proton pump inhibitors in terms of *Helicobacter pylori* eradication? Gut Liver. 2021;15(6):799‐810. doi:10.5009/gnl20242 33850058 PMC8593510

[10] Sarnaik MK , Modi S , Pisipati Y , et al. Proton pump inhibitors: exploring cardiovascular complications and prescription protocol. Cureus. 2021;13(7):e16744. doi:10.7759/cureus.16744 34354892 PMC8328806

[11] Gu L , Guan J , Huang Z , et al. β‐Cyclodextrin covalent organic framework supported by polydopamine as stationary phases for electrochromatographic enantioseparation. Electrophoresis. 2022;121:21‐25. doi:10.1002/elps.202200029 35353923

[12] Hammond CL , Roztocil E , Gupta V , Feldon SE , Woeller CF . More than meets the eye: the aryl hydrocarbon receptor is an environmental sensor, physiological regulator and a therapeutic target in ocular disease. Front Toxicol. 2022;3(4):791082. doi:10.3389/ftox.2022.791082 PMC8915869 35295218

[13] Wang K , Lou D , Dai W , Fu R , Ma Z . Comparison of sequential therapy with concomitant therapy in first‐line treatment of *Helicobacter pylori*: an updated meta‐analysis. J Med Microbiol. 2022;71(1):845‐847. doi:10.1099/jmm.0.001490 35041577

[14] Chen Z , Zheng L , Chen Y , et al. Loss of ubiquitin‐specific peptidase 18 destabilizes 14‐3‐3ζ protein and represses lung cancer metastasis. Cancer Biol Ther. 2022;23(1):265‐280. doi:10.1080/15384047.2022.2054242 35387560 PMC8993103

[15] Zhang X , Liu Q , Zhang T , et al. Bone‐targeted nanoplatform enables efficient modulation of bone tumor microenvironment for prostate cancer bone metastasis treatment. Drug Deliv. 2022;29(1):889‐905. doi:10.1080/10717544.2022.2050845 35285760 PMC8928789

[16] Wang D , Wan Z , Yang Q , et al. Sonodynamical reversion of immunosuppressive microenvironment in prostate cancer via engineered exosomes. Drug Deliv. 2022;29(1):702‐713. doi:10.1080/10717544.2022.2044937 35236203 PMC8903759

[17] Wang T , Zhang H , Qiu W , Han Y , Liu H , Li Z . Biomimetic nanoparticles directly remodel immunosuppressive microenvironment for boosting glioblastoma immunotherapy. Bioact Mater. 2022;5(16):418‐432. doi:10.1016/j.bioactmat.2021.12.029 PMC8965726 35386309

[18] Ostrowski RP , Pucko EB . Harnessing oxidative stress for anti‐glioma therapy. Neurochem Int. 2022 Mar;154:105281. doi:10.1016/j.neuint.2022.105281 35038460

[19] Uyar R . Glioblastoma microenvironment: the stromal interactions. Pathol Res Pract. 2022;232:153813. doi:10.1016/j.prp.2022.153813 35228161

[20] Salami R , Salami M , Mafi A , Vakili O , Asemi Z . Circular RNAs and glioblastoma multiforme: focus on molecular mechanisms. Cell Commun Signal. 2022;20(1):13. doi:10.1186/s12964-021-00809-9 35090496 PMC8796413

[21] Hu S , Kao HY , Yang T , Wang Y . Early and bi‐hemispheric seizure onset in a rat glioblastoma multiforme model. Neurosci Lett. 2022;766:136351. doi:10.1016/j.neulet.2021.136351 34793898 PMC8642883

[22] Sporikova Z , Slavkovsky R , Tuckova L , et al. IDH1/2 mutations in patients with diffuse gliomas: a single centre retrospective massively parallel sequencing analysis. Appl Immunohistochem Mol Morphol. 2022;30(3):178‐183. doi:10.1097/PAI.0000000000000997 35262523 PMC8920008

[23] Fleischmann DF , Schön R , Corradini S , et al. Multifocal high‐grade glioma radiotherapy safety and efficacy. Radiat Oncol. 2021;16(1):165. doi:10.1186/s13014-021-01886-3 34454558 PMC8400399

[24] Wang Y , Bao G , Zhang M , et al. CRB2 enhances malignancy of glioblastoma via activation of the NF‐κB pathway. Exp Cell Res. 2022;414(1):113077. doi:10.1016/j.yexcr.2022.113077 35219647

[25] Trivieri N , Visioli A , Mencarelli G , et al. Growth factor independence underpins a paroxysmal, aggressive Wnt5a^High^/EphA2^Low^ phenotype in glioblastoma stem cells, conducive to experimental combinatorial therapy. J Exp Clin Cancer Res. 2022;41(1):139. doi:10.1186/s13046-022-02333-1 35414102 PMC9004109

[26] Kurokawa R , Baba A , Emile P , et al. Neuroimaging features of angiocentric glioma: a case series and systematic review. J Neuroimaging. 2022;32:389‐399. doi:10.1111/jon.12983 35201652 PMC9306893

[27] Yang K , Wu Z , Zhang H , et al. Glioma targeted therapy: insight into future of molecular approaches. Mol Cancer. 2022;21(1):39. doi:10.1186/s12943-022-01513-z 35135556 PMC8822752

[28] Gritsch S , Batchelor TT , Gonzalez Castro LN . Diagnostic, therapeutic, and prognostic implications of the 2021 World Health Organization classification of tumors of the central nervous system. Cancer. 2022;128(1):47‐58. doi:10.1002/cncr.33918 34633681

[29] Lin Y , Sun H , Dang Y , Li Z . Isoliquiritigenin inhibits the proliferation and induces the differentiation of human glioma stem cells. Oncol Rep. 2018;39(2):687‐694. doi:10.3892/or.2017.6154 29251326

[30] Alanazi R , Nakatogawa H , Wang H , et al. Inhibition of TRPM7 with carvacrol suppresses glioblastoma functions in vivo. Eur J Neurosci. 2022;55(6):1483‐1491. doi:10.1111/ejn.15647 35277895

[31] Valiente M , Obenauf AC , Jin X , et al. Serpins promote cancer cell survival and vascular co‐option in brain metastasis. Cell. 2014;156(5):1002‐1016. doi:10.1016/j.cell.2014.01.040 24581498 PMC3988473

[32] Crotty EE , Smith SMC , Brasel K , et al. Medulloblastoma recurrence and metastatic spread are independent of colony‐stimulating factor 1 receptor signaling and macrophage survival. J Neurooncol. 2021;153(2):225‐237. doi:10.1007/s11060-021-03767-x 33963961 PMC8248272

[33] Wang R , Wang X , Li J , Di L , Zhou J , Ding Y . Lipoprotein‐biomimetic nanostructure enables tumor‐targeted penetration delivery for enhanced photo‐gene therapy towards glioma. Bioact Mater. 2021;2(13):286‐299. doi:10.1016/j.bioactmat.2021.10.039 PMC8844848 35224309

[34] Gable A , Fiore B , Cheatham J . Clarification of eosinophilic esophagitis treatment in the DoD retention standards. Mil Med. 2022;187(1–2):35‐36. doi:10.1093/milmed/usab359 34453177

[35] Arnoux A , Bailhache M , Tetard C , et al. Proton pump inhibitors are still overprescribed for hospitalized children. Arch Pediatr. 2022;29(4):258‐262. doi:10.1016/j.arcped.2022.02.004 35304031

[36] Singh G , Haileselassie Y , Briscoe L , et al. The effect of gastric acid suppression on probiotic colonization in a double blinded randomized clinical trial. Clin Nutr ESPEN. 2022;47:70‐77. doi:10.1016/j.clnesp.2021.11.005 35063245

[37] Chung CS , Chen CC , Chen KC , et al. Randomized controlled trial of early endoscopy for upper gastrointestinal bleeding in acute coronary syndrome patients. Sci Rep. 2022;12(1):5798. doi:10.1038/s41598-022-09911-5 35388113 PMC8986851

[38] Saven H , Zhong L , McFarlane IM . Co‐prescription of dual‐antiplatelet therapy and proton pump inhibitors: current guidelines. Cureus. 2022;14(2):e21885. doi:10.7759/cureus.21885 35273851 PMC8901154

[39] Cheng FC , Ho YF , Hung LC , Chen CF , Tsai TH . Determination and pharmacokinetic profile of omeprazole in rat blood, brain and bile by microdialysis and high‐performance liquid chromatography. J Chromatogr A. 2002;949(1–2):35‐42. doi:10.1016/s0021-9673(01)01225-0 11999751

[40] Rojo LE , Alzate‐Morales J , Saavedra IN , Davies P , Maccioni RB . Selective interaction of lansoprazole and astemizole with tau polymers: potential new clinical use in diagnosis of Alzheimer's disease. J Alzheimers Dis. 2010;19(2):573‐589. doi:10.3233/JAD-2010-1262 20110603 PMC2951486

[41] Miner P Jr , Katz PO , Chen Y , Sostek M . Gastric acid control with esomeprazole, lansoprazole, omeprazole, pantoprazole, and rabeprazole: a five‐way crossover study. Am J Gastroenterol. 2003;98(12):2616‐2620. doi:10.1111/j.1572-0241.2003.08783.x 14687806

[42] Grattagliano I , Portincasa P , Mastronardi M , Palmieri VO , Palasciano G . Esomeprazole‐induced central fever with severe myalgia. Ann Pharmacother. 2005;39(4):757‐760. doi:10.1345/aph.1E377 15741426

[43] Papp LA , Hancu G , Kelemen H , Tóth G . Chiral separation in the class of proton pump inhibitors by chromatographic and electromigration techniques: an overview. Electrophoresis. 2021;42(17–18):1761‐1789. doi:10.1002/elps.202100032 34004039

[44] Bruno G , Zaccari P , Rocco G , et al. Proton pump inhibitors and dysbiosis: current knowledge and aspects to be clarified. World J Gastroenterol. 2019;25(22):2706‐2719. doi:10.3748/wjg.v25.i22.2706 31235994 PMC6580352

[45] Wang L , Chai Y , Zhu W , Pan Y , Sun C , Zeng S . Doubly charged trimeric cluster ions: effective in mutual chiral recognition of tadalafil and three proton pump inhibitors. Analyst. 2017;142(5):745‐751. doi:10.1039/c6an02666d 28197557

[46] Seidel T , Scholl S , Krebs M , et al. Regulation of the V‐type ATPase by redox modulation. Biochem J. 2012;448(2):243‐251. doi:10.1042/BJ20120976 22943363

[47] Makunts T , Alpatty S , Lee KC , Atayee RS , Abagyan R . Proton‐pump inhibitor use is associated with a broad spectrum of neurological adverse events including impaired hearing, vision, and memory. Sci Rep. 2019;9(1):17280. doi:10.1038/s41598-019-53622-3 31754136 PMC6872761

[48] Chen YC , Backus KM , Merkulova M , et al. Covalent Modulators of the Vacuolar ATPase. J Am Chem Soc. 2017;139(2):639‐642. doi:10.1021/jacs.6b12511 28010062 PMC5274637

[49] Yuan J , Liu W , Karvar S , et al. Potassium channel KCNJ15 is required for histamine‐stimulated gastric acid secretion. Am J Physiol Cell Physiol. 2015;309(4):C264‐C270. doi:10.1152/ajpcell.00012.2015 26108660

[50] He J , Shi XY , Li ZM , et al. Proton pump inhibitors can reverse the YAP mediated paclitaxel resistance in epithelial ovarian cancer. BMC Mol Cell Biol. 2019;20(1):49. doi:10.1186/s12860-019-0227-y 31718559 PMC6852784

[51] Lee YY , Jeon HK , Hong JE , et al. Proton pump inhibitors enhance the effects of cytotoxic agents in chemoresistant epithelial ovarian carcinoma. Oncotarget. 2015;6(33):35040‐35050. doi:10.18632/oncotarget.5319 26418900 PMC4741507

[52] Ihraiz WG , Ahram M , Bardaweel SK . Proton pump inhibitors enhance chemosensitivity, promote apoptosis, and suppress migration of breast cancer cells. Acta Pharm. 2020;70(2):179‐190. doi:10.2478/acph-2020-0020 31955147

[53] Altundag K . Letter comments on: drug‐drug interactions between palbociclib and proton pump inhibitors may significantly affect clinical outcome of patients with metastatic breast cancer. ESMO Open. 2022;7(1):100382. doi:10.1016/j.esmoop.2022.100382 35144122 PMC8844652

[54] Wang J , Shan F , Li S , Li Z , Wu Q . Effect of administration of a proton pump inhibitor for ulcerative differentiated early gastric cancer prior to endoscopic submucosal dissection. Dig Endosc. 2021;33(6):939‐947. doi:10.1111/den.13892 33184984

[55] Jin UH , Lee SO , Pfent C , Safe S . The aryl hydrocarbon receptor ligand omeprazole inhibits breast cancer cell invasion and metastasis. BMC Cancer. 2014;9(14):498. doi:10.1186/1471-2407-14-498 PMC4226953 25011475

[56] Wang CJ , Li D , Danielson JA , et al. Proton pump inhibitors suppress DNA damage repair and sensitize treatment resistance in breast cancer by targeting fatty acid synthase. Cancer Lett. 2021;509:1‐12. doi:10.1016/j.canlet.2021.03.026 33813001 PMC8167934

[57] Menter T , Tzankov A , Dirnhofer S . The tumor microenvironment of lymphomas: insights into the potential role and modes of actions of checkpoint inhibitors. Hematol Oncol. 2021;39(1):3‐10. 33105031 10.1002/hon.2821

[58] Asadirad A , Baghaei K , Hashemi SM , et al. Dendritic cell immunotherapy with miR‐155 enriched tumor‐derived exosome suppressed cancer growth and induced antitumor immune responses in murine model of colorectal cancer induced by CT26 cell line. Int Immunopharmacol. 2022;104:108493. doi:10.1016/j.intimp.2021.108493 35032826

[59] Morgan D , Berggren KL , Spiess CD , et al. Mitogen‐activated protein kinase‐activated protein kinase‐2 (MK2) and its role in cell survival, inflammatory signaling, and migration in promoting cancer. Mol Carcinog. 2022;61(2):173‐199. doi:10.1002/mc.23348 34559922 PMC8799529

[60] Annese T , Tamma R , Bozza M , Zito A , Ribatti D . Autocrine/paracrine loop between SCF^+^/c‐kit^+^ mast cells promotes cutaneous melanoma progression. Front Immunol. 2022;24(13):794974. doi:10.3389/fimmu.2022.794974 PMC8818866 35140718

[61] Wang H , Yung MMH , Ngan HYS , Chan KKL , Chan DW . The impact of the tumor microenvironment on macrophage polarization in cancer metastatic progression. Int J Mol Sci. 2021;22(12):6560. doi:10.3390/ijms22126560 34207286 PMC8235734

[62] Demuynck R , Efimova I , Naessens F , Krysko DV . Immunogenic ferroptosis and where to find it? J Immunother Cancer. 2021;9(12):e003430. doi:10.1136/jitc-2021-003430 34903554 PMC8671998

[63] Campion KL , McCormick WD , Warwicker J , et al. Pathophysiologic changes in extracellular pH modulate parathyroid calcium‐sensing receptor activity and secretion via a histidine‐independent mechanism. J Am Soc Nephrol. 2015;26(9):2163‐2171. doi:10.1681/ASN.2014070653 25556167 PMC4552114

[64] Weijenborg PW , Smout AJ , Bredenoord AJ . Esophageal acid sensitivity and mucosal integrity in patients with functional heartburn. Neurogastroenterol Motil. 2016;28(11):1649‐1654. doi:10.1111/nmo.12864 27194216

[65] Cao L , Li W , Yang X , et al. Inhibition of host Ogr1 enhances effector CD8^+^ T‐cell function by modulating acidic microenvironment. Cancer Gene Ther. 2021;28(10–11):1213‐1224. doi:10.1038/s41417-021-00354-0 34158625 PMC8571096

[66] Gribben JG , Fowler N , Morschhauser F . Mechanisms of action of lenalidomide in B‐cell non‐Hodgkin lymphoma. J Clin Oncol. 2015;33(25):2803‐2811. doi:10.1200/JCO.2014.59.5363 26195701 PMC5320950

[67] Spugnini EP , Sonveaux P , Stock C , et al. Proton channels and exchangers in cancer. Biochim Biophys Acta. 2015;1848(10 Pt B):2715‐2726. doi:10.1016/j.bbamem.2014.10.015 25449995

[68] Xu X , Fei J , Xu Y , et al. Boric acid‐fueled ATP synthesis by F_o_ F_1_ ATP synthase reconstituted in a supramolecular architecture. Angew Chem Int Ed Engl. 2021;60(14):7617‐7620. doi:10.1002/anie.202016253 33369011

[69] Liu J , Wang Y , Zhang M , et al. Color fading in lotus (Nelumbo nucifera) petals is manipulated both by anthocyanin biosynthesis reduction and active degradation. Plant Physiol Biochem. 2022;15(179):100‐107. doi:10.1016/j.plaphy.2022.03.021 35325657

[70] Halcrow P , Datta G , Ohm JE , Soliman ML , Chen X , Geiger JD . Role of endolysosomes and pH in the pathogenesis and treatment of glioblastoma. Cancer Rep. 2019;2(6):e1177. doi:10.1002/cnr2.1177 32095788 PMC7039640

[71] Asgharzadeh MR , Barar J , Pourseif MM , et al. Molecular machineries of pH dysregulation in tumor microenvironment: potential targets for cancer therapy. Bioimpacts. 2017;7(2):115‐133. doi:10.15171/bi.2017.15 28752076 PMC5524986

[72] Yu C , Liu H , Guo C , et al. Dextran sulfate‐based MMP‐2 enzyme‐sensitive SR‐A receptor targeting nanomicelles for the treatment of rheumatoid arthritis. Drug Deliv. 2022;29(1):454‐465. doi:10.1080/10717544.2022.2032482 35119317 PMC8855847

[73] Wi DH , Cha JH , Jung YS . Mucin in cancer: a stealth cloak for cancer cells. BMB Rep. 2021;54(7):344‐355. doi:10.5483/BMBRep.2021.54.7.064 34154702 PMC8328826

[74] Ali A , Biswas R , Bhattacharjee S , Nath P , Pan S , Bagchi A . Comparative analyses of the relative effects of various mutations in major histocompatibility complex I‐a way to predict protein‐protein interactions. Appl Biochem Biotechnol. 2016;180(1):152‐164. doi:10.1007/s12010-016-2090-z 27125960

[75] Choi SJ , Cho H , Yea K , Baek MC . Immune cell‐derived small extracellular vesicles in cancer treatment. BMB Rep. 2021;54:335‐343. 34353429 10.5483/BMBRep.2022.55.1.133 PMC8810553

[76] Chadet S , Allard J , Brisson L , et al. P2x4 receptor promotes mammary cancer progression by sustaining autophagy and associated mesenchymal transition. Oncogene. 2022;9(8):432‐445. doi:10.1038/s41388-022-02297-8 35411034

[77] Cao Y , Chen M , Tang D , et al. The proton pump inhibitor pantoprazole disrupts protein degradation systems and sensitizes cancer cells to death under various stresses. Cell Death Dis. 2018;9(6):604. doi:10.1038/s41419-018-0642-6 29789637 PMC5964200

[78] Nam LB , Keum YS . Regulation of NRF2 by Na^+^/K^+^‐ATPase: implication of tyrosine phosphorylation of Src. Free Radic Res. 2020;54(11–12):883‐893. doi:10.1080/10715762.2020.1735633 32114856

[79] Santos‐Pereira C , Rodrigues LR , Côrte‐Real M . Emerging insights on the role of V‐ATPase in human diseases: therapeutic challenges and opportunities. Med Res Rev. 2021;41(4):1927‐1964. doi:10.1002/med.21782 33483985

[80] Chiang YR , Wang LC , Lin HT , Lin JH . Bioactivity of orange‐spotted grouper (Epinephelus coioides) cathepsin L: proteolysis of bacteria and regulation of the innate immune response. Fish Shellfish Immunol. 2022;122:399‐408. doi:10.1016/j.fsi.2022.02.003 35176469

[81] Krueger F , Kappert K , Foryst‐Ludwig A , et al. AT1‐receptor blockade attenuates outward aortic remodeling associated with diet‐induced obesity in mice. Clin Sci (Lond). 2017;131(15):1989‐2005. doi:10.1042/CS20170131 28646121

[82] Chae YC , Vaira V , Caino MC , et al. Mitochondrial Akt regulation of hypoxic tumor reprogramming. Cancer Cell. 2016;30(2):257‐272. doi:10.1016/j.ccell.2016.07.004 27505672 PMC5131882

[83] Ordway B , Gillies RJ , Damaghi M . Extracellular acidification induces lysosomal dysregulation. Cells. 2021;10(5):1188. doi:10.3390/cells10051188 34067971 PMC8152284

[84] Nath B , Bidkar AP , Kumar V , et al. Deciphering hydrodynamic and drug‐resistant behaviors of metastatic EMT breast cancer cells moving in a constricted microcapillary. J Clin Med. 2019;8(8):1194. doi:10.3390/jcm8081194 31404980 PMC6722803

[85] Wang G , Zhou X , Guo Z , et al. The anti‐fibrosis drug Pirfenidone modifies the immunosuppressive tumor microenvironment and prevents the progression of renal cell carcinoma by inhibiting tumor autocrine TGF‐β. Cancer Biol Ther. 2022;23(1):150‐162. doi:10.1080/15384047.2022.2035629 35130111 PMC8824226

[86] Cao X , Yang Q , Qin J , et al. V‐ATPase promotes transforming growth factor‐β‐induced epithelial‐mesenchymal transition of rat proximal tubular epithelial cells. Am J Physiol Renal Physiol. 2012;302(9):F1121‐F1132. doi:10.1152/ajprenal.00278.2011 22129967

[87] Zhang Y , Yang F , Peng X , et al. Hypoxia constructing the prognostic model of colorectal adenocarcinoma and related to the immune microenvironment. Front Cell Dev Biol. 2021;20(9):665364. doi:10.3389/fcell.2021.665364 PMC8093637 33959617

[88] Lefebvre TL , Brown E , Hacker L , et al. The potential of photoacoustic imaging in radiation oncology. Front Oncol. 2022;3(12):803777. doi:10.3389/fonc.2022.803777 PMC8928467 35311156

[89] Wang M , Yan J , Cao X , Hua P , Li Z . Hydrogen sulfide modulates epithelial‐mesenchymal transition and angiogenesis in non‐small cell lung cancer via HIF‐1α activation. Biochem Pharmacol. 2020;172:113775. doi:10.1016/j.bcp.2019.113775 31870768

[90] Campbell EJ , Dachs GU , Morrin HR , Davey VC , Robinson BA , Vissers MCM . Activation of the hypoxia pathway in breast cancer tissue and patient survival are inversely associated with tumor ascorbate levels. BMC Cancer. 2019;19(1):307. doi:10.1186/s12885-019-5503-x 30943919 PMC6448303

[91] Trivlidis J , Aloufi N , Al‐Habeeb F , et al. HuR drives lung fibroblast differentiation but not metabolic reprogramming in response to TGF‐β and hypoxia. Respir Res. 2021;22(1):323. doi:10.1186/s12931-021-01916-4 34963461 PMC8715577

[92] Man CH , Mercier FE , Liu N , et al. Proton export alkalinizes intracellular pH and reprograms carbon metabolism to drive normal and malignant cell growth. Blood. 2022;139(4):502‐522. doi:10.1182/blood.2021011563 34610101 PMC8796654

[93] Michl J , Wang Y , Monterisi S , et al. CRISPR‐Cas9 screen identifies oxidative phosphorylation as essential for cancer cell survival at low extracellular pH. Cell Rep. 2022;38(10):110493. doi:10.1016/j.celrep.2022.110493 35263578 PMC8924371

[94] Goetze K , Fabian CG , Siebers A , et al. Manipulation of tumor metabolism for therapeutic approaches: ovarian cancer‐derived cell lines as a model system. Cell Oncol (Dordr). 2015;38(5):377‐385. doi:10.1007/s13402-015-0237-5 26288178 PMC13004272

[95] Gao L , Xu QH , Ma LN , et al. Trophoblast‐derived lactic acid orchestrates Decidual macrophage differentiation via SRC/LDHA signaling in early pregnancy. Int J Biol Sci. 2022;18(2):599‐616. doi:10.7150/ijbs.67816 35002512 PMC8741856

[96] Morimoto C , Takedachi M , Kawasaki K , et al. Hypoxia stimulates collagen hydroxylation in gingival fibroblasts and periodontal ligament cells. J Periodontol. 2021;92(11):1635‐1645. doi:10.1002/JPER.20-0670 33660864

[97] Ji RC . Hypoxia and lymphangiogenesis in tumor microenvironment and metastasis. Cancer Lett. 2014;346(1):6‐16. doi:10.1016/j.canlet.2013.12.001 24333723

[98] Gorick CM , Saucerman JJ , Price RJ . Computational model of brain endothelial cell signaling pathways predicts therapeutic targets for cerebral pathologies. J Mol Cell Cardiol. 2022;164:17‐28. doi:10.1016/j.yjmcc.2021.11.005 34798125 PMC8958390

[99] Cantó A , Olivar T , Romero FJ , Miranda M . Nitrosative stress in retinal pathologies: review. Antioxidants (Basel). 2019;8(11):543. doi:10.3390/antiox8110543 31717957 PMC6912788

[100] Pagnotta S , Tramutola A , Barone E , et al. CAPE and its synthetic derivative VP961 restore BACH1/NRF2 axis in down syndrome. Free Radic Biol Med. 2022;183:1‐13. doi:10.1016/j.freeradbiomed.2022.03.006 35283228

[101] Zhou W , Chen C , Shi Y , et al. Targeting glioma stem cell‐derived Pericytes disrupts the blood‐tumor barrier and improves chemotherapeutic efficacy. Cell Stem Cell. 2017;21(5):591‐603.e4. doi:10.1016/j.stem.2017.10.002 29100012 PMC5687837

[102] Daneman R , Prat A . The blood‐brain barrier. Cold Spring Harb Perspect Biol. 2015;7(1):a020412. doi:10.1101/cshperspect.a020412 25561720 PMC4292164

[103] Prados MD . Future directions in the treatment of malignant gliomas with temozolomide. Semin Oncol. 2000;27(3 Suppl 6):41‐46. 10866349

[104] Munoz JL , Walker ND , Scotto KW , Rameshwar P . Temozolomide competes for P‐glycoprotein and contributes to chemoresistance in glioblastoma cells. Cancer Lett. 2015;367(1):69‐75. doi:10.1016/j.canlet.2015.07.013 26208431

[105] Park SH , Kim MJ , Jung HH , et al. Safety and feasibility of multiple blood‐brain barrier disruptions for the treatment of glioblastoma in patients undergoing standard adjuvant chemotherapy. J Neurosurg. 2020;3:1‐9. doi:10.3171/2019.10.JNS192206 31899873

[106] Declèves X , Amiel A , Delattre JY , Scherrmann JM . Role of ABC transporters in the chemoresistance of human gliomas. Curr Cancer Drug Targets. 2006;6(5):433‐445. doi:10.2174/156800906777723930 16918310

[107] Yang G , Xu S , Peng L , Li H , Zhao Y , Hu Y . The hypoxia‐mimetic agent CoCl₂ induces chemotherapy resistance in LOVO colorectal cancer cells. Mol Med Rep. 2016;13(3):2583‐2589. doi:10.3892/mmr.2016.4836 26846577 PMC4768964

[108] Kachalaki S , Ebrahimi M , Mohamed Khosroshahi L , Mohammadinejad S , Baradaran B . Cancer chemoresistance; biochemical and molecular aspects: a brief overview. Eur J Pharm Sci. 2016;30(89):20‐30. doi:10.1016/j.ejps.2016.03.025 27094906

[109] Zhou X , Jin N , Chen B . Tetrandrine overcomes drug resistance mediated by bone marrow microenvironment by regulating the expression of P‐glycoprotein in acute leukemia. Hematology. 2022;27(1):274‐279. doi:10.1080/16078454.2022.2034256 35192780

[110] Uprety B , Chandran R , Arderne C , Abrahamse H . Anticancer activity of urease mimetic cobalt (III) complexes on A549‐lung cancer cells: targeting the acidic microenvironment. Pharmaceutics. 2022;14(1):211. doi:10.3390/pharmaceutics14010211 35057107 PMC8780642

[111] Charifson PS , Walters WP . Acidic and basic drugs in medicinal chemistry: a perspective. J Med Chem. 2014;57(23):9701‐9717. doi:10.1021/jm501000a 25180901

[112] Hraběta J , Belhajová M , Šubrtová H , Merlos Rodrigo MA , Heger Z , Eckschlager T . Drug sequestration in lysosomes as one of the mechanisms of chemoresistance of cancer cells and the possibilities of its inhibition. Int J Mol Sci. 2020;21(12):4392. doi:10.3390/ijms21124392 32575682 PMC7352242

[113] Tsuruo T , Naito M , Tomida A , et al. Molecular targeting therapy of cancer: drug resistance, apoptosis and survival signal. Cancer Sci. 2003 Jan;94(1):15‐21. doi:10.1111/j.1349-7006.2003.tb01345.x 12708468 PMC11160265

[114] Kanwore K , Kanwore K , Adzika GK , et al. Cancer metabolism: the role of immune cells epigenetic alteration in tumorigenesis, progression, and metastasis of glioma. Front Immunol. 2022;22(13):831636. doi:10.3389/fimmu.2022.831636 PMC8980436 35392088

[115] Zhao Z , Wang Z , Wu Y , Liao D , Zhao B . Comprehensive analysis of TAMs marker genes in glioma for predicting prognosis and immunotherapy response. Mol Immunol. 2022;144:78‐95. doi:10.1016/j.molimm.2022.02.012 35203024

[116] Strepkos D , Markouli M , Klonou A , Piperi C , Papavassiliou AG . Insights in the immunobiology of glioblastoma. J Mol Med (Berl). 2020;98(1):1‐10. doi:10.1007/s00109-019-01835-4 31650201

[117] Mummolo S , Botticelli G , Quinzi V , Giuca G , Mancini L , Marzo G . Implant‐safe test in patients with peri‐implantitis. J Biol Regul Homeost Agents. 2020;34(3 Suppl. 1):147‐153. 32618172

[118] Rong X , Huang B , Qiu S , Li X , He L , Peng Y . Tumor‐associated macrophages induce vasculogenic mimicry of glioblastoma multiforme through cyclooxygenase‐2 activation. Oncotarget. 2016;7(51):83976‐83986. 27824617 10.18632/oncotarget.6930 PMC5356639

[119] Zhu D , Li Y , Zhang Z , et al. Recent advances of nanotechnology‐based tumor vessel‐targeting strategies. J Nanobiotechnology. 2021;19(1):435. doi:10.1186/s12951-021-01190-y 34930293 PMC8686559

[120] Okikawa S , Morine Y , Saito Y , et al. Inhibition of the VEGF signaling pathway attenuates tumor‐associated macrophage activity in liver cancer. Oncol Rep. 2022;47(4):71. doi:10.3892/or.2022.8282 35169858 PMC8867251

[121] Yang Y , Zhang Y , Chen X , Su Z , Deng Y , Zhao Q . Khasianine ameliorates psoriasis‐like skin inflammation and represses TNF‐α/NF‐κB axis mediated transactivation of IL‐17A and IL‐33 in keratinocytes. J Ethnopharmacol. 2022;28(292):115124. doi:10.1016/j.jep.2022.115124 35183690

[122] Tamai S , Ichinose T , Tsutsui T , et al. Tumor microenvironment in glioma invasion. Brain Sci. 2022;12(4):505. doi:10.3390/brainsci12040505 35448036 PMC9031400

[123] Ermakov MS , Nushtaeva AA , Richter VA , Koval OA . Cancer‐associated fibroblasts and their role in tumor progression. Vavilovskii Zhurnal Genet Selektsii. 2022;26(1):14‐21. doi:10.18699/VJGB-22-03 35342854 PMC8894099

[124] Zhang Y , Bian Y , Wang Y , et al. HIF‐1α is necessary for activation and tumour‐promotion effect of cancer‐associated fibroblasts in lung cancer. J Cell Mol Med. 2021;25(12):5457‐5469. doi:10.1111/jcmm.16556 33943003 PMC8184678

[125] Zimmerlin L , Park TS , Zambidis ET , Donnenberg VS , Donnenberg AD . Mesenchymal stem cell secretome and regenerative therapy after cancer. Biochimie. 2013;95(12):2235‐2245. doi:10.1016/j.biochi.2013.05.010 23747841 PMC3825748

[126] Czekay RP , Cheon DJ , Samarakoon R , Kutz SM , Higgins PJ . Cancer‐associated fibroblasts: mechanisms of tumor progression and novel therapeutic targets. Cancers (Basel). 2022;14(5):1231. doi:10.3390/cancers14051231 35267539 PMC8909913

[127] Paolino G , Corsetti P , Moliterni E , et al. Mast cells and cancer. G Ital Dermatol Venereol. 2019 Dec;154(6):650‐668. doi:10.23736/S0392-0488.17.05818-7 29192477

[128] Abiko K , Hayashi T , Yamaguchi K , Mandai M , Konishi I . Potential novel ovarian cancer treatment targeting myeloid‐derived suppressor cells. Cancer Invest. 2021;39(4):310‐314. doi:10.1080/07357907.2020.1871487 33428503

[129] Alghamri MS , McClellan BL , Hartlage MS , et al. Targeting neuroinflammation in brain cancer: uncovering mechanisms, pharmacological targets, and Neuropharmaceutical developments. Front Pharmacol. 2021;18(12):680021. doi:10.3389/fphar.2021.680021 PMC8167057 34084145

[130] Abbas S , Singh SK , Saxena AK , Tiwari S , Sharma LK , Tiwari M . Role of autophagy in regulation of glioma stem cells population during therapeutic stress. J Stem Cells Regen Med. 2020;16(2):80‐89. doi:10.46582/jsrm.1602012 33414584 PMC7772813

[131] Rakesh R , LC PD , Sakthivel KM , Rasmi RR . Role and regulation of autophagy in cancer. Biochim Biophys Acta Mol Basis Dis. 2022;1868(7):166400. doi:10.1016/j.bbadis.2022.166400 35341960

[132] Yu Y , Liu B , Li X , et al. ATF4/CEMIP/PKCα promotes anoikis resistance by enhancing protective autophagy in prostate cancer cells. Cell Death Dis. 2022;13(1):46. doi:10.1038/s41419-021-04494-x 35013120 PMC8748688

[133] Kim J , Chee WY , Yabuta N , Kajiwara K , Nada S , Okada M . Atg5‐mediated autophagy controls apoptosis/anoikis via p53/Rb pathway in naked mole‐rat fibroblasts. Biochem Biophys Res Commun. 2020;528(1):146‐153. doi:10.1016/j.bbrc.2020.05.083 32451084

[134] Li Y , Gao S , Du X , Ji J , Xi Y , Zhai G . Advances in autophagy as a target in the treatment of tumours. J Drug Target. 2022;30(2):166‐187. doi:10.1080/1061186X.2021.1961792 34319838

[135] He C , Lu S , Wang XZ , et al. FOXO3a protects glioma cells against temozolomide‐induced DNA double strand breaks via promotion of BNIP3‐mediated mitophagy. Acta Pharmacol Sin. 2021;42(8):1324‐1337. doi:10.1038/s41401-021-00663-y 33879840 PMC8285492

[136] Yuan T , Wang J , Shi C , et al. Downregulation of FAPP2 gene induces cell autophagy and inhibits PI3K/AKT/mTOR pathway in T‐cell acute lymphoblastic leukemia. Hematol Oncol. 2022;40(2):249‐257. doi:10.1002/hon.2948 34796518

[137] Duggan MR , Weaver M , Khalili K . PAM (PIK3/AKT/mTOR) signaling in glia: potential contributions to brain tumors in aging. Aging (Albany NY). 2021;13(1):1510‐1527. doi:10.18632/aging.202459 33472174 PMC7835031

[138] Trelford CB , Di Guglielmo GM . Canonical and non‐canonical TGFβ signaling activate autophagy in an ULK1‐dependent manner. Front Cell Dev Biol. 2021;25(9):712124. doi:10.3389/fcell.2021.712124 PMC8573198 34760883

[139] Lu G , Wu Z , Shang J , Xie Z , Chen C , Zhang C . The effects of metformin on autophagy. Biomed Pharmacother. 2021 May;137:111286. doi:10.1016/j.biopha.2021.111286 33524789

[140] Park JW , Jeong J , Bae YS . Protein kinase CK2 is upregulated by calorie restriction and induces autophagy. Mol Cells. 2022;45(3):112‐121. doi:10.14348/molcells.2021.0183 34949740 PMC8926869

[141] Li Y , Li S , Wu H . Ubiquitination‐proteasome system (UPS) and autophagy two Main protein degradation machineries in response to cell stress. Cell. 2022;11(5):851. doi:10.3390/cells11050851 PMC8909305 35269473

[142] Chipurupalli S , Ganesan R , Martini G , et al. Cancer cells adapt FAM134B/BiP mediated ER‐phagy to survive hypoxic stress. Cell Death Dis. 2022;13(4):357. doi:10.1038/s41419-022-04813-w 35436985 PMC9016075

[143] Qin X , Wang J , Chen S , et al. Astrocytic p75^NTR^ expression provoked by ischemic stroke exacerbates the blood‐brain barrier disruption. Glia. 2022;70(5):892‐912. doi:10.1002/glia.24146 35064700

[144] Izdebska M , Zielińska W , Krajewski A , et al. Downregulation of MMP‐9 enhances the anti‐migratory effect of cyclophosphamide in MDA‐MB‐231 and MCF‐7 breast cancer cell lines. Int J Mol Sci. 2021;22(23):12783. doi:10.3390/ijms222312783 34884588 PMC8657655

[145] Ahir BK , Engelhard HH , Lakka SS . Tumor development and angiogenesis in adult brain tumor: glioblastoma. Mol Neurobiol. 2020;57(5):2461‐2478. doi:10.1007/s12035-020-01892-8 32152825 PMC7170819

[146] Cheng B , Hong X , Wang L , et al. Curzerene suppresses progression of human glioblastoma through inhibition of glutathione S‐transferase A4. CNS Neurosci Ther. 2022;28(5):690‐702. doi:10.1111/cns.13800 35048517 PMC8981481

[147] Yang B , Ding L , Chen Y , Shi J . Augmenting tumor‐starvation therapy by cancer cell autophagy inhibition. Adv Sci (Weinh). 2020;7(6):1902847. doi:10.1002/advs.201902847 32195096 PMC7080508

[148] Molinaro AM , Taylor JW , Wiencke JK , Wrensch MR . Genetic and molecular epidemiology of adult diffuse glioma. Nat Rev Neurol. 2019;15(7):405‐417. doi:10.1038/s41582-019-0220-2 31227792 PMC7286557

[149] Jiang L , Liu J . Immunological effect of tyrosine kinase inhibitors on the tumor immune environment in non‐small cell lung cancer. Oncol Lett. 2022;23(5):165. doi:10.3892/ol.2022.13285 35414830 PMC8988264

[150] Geeviman K , Babu D , Prakash BP . Pantoprazole induces mitochondrial apoptosis and attenuates NF‐κB signaling in glioma cells. Cell Mol Neurobiol. 2018;38(8):1491‐1504. doi:10.1007/s10571-018-0623-4 30302629 PMC11469912

[151] Shen W , Zou X , Chen M , et al. Effect of pantoprazole on human gastric adenocarcinoma SGC7901 cells through regulation of phospho‐LRP6 expression in Wnt/β‐catenin signaling. Oncol Rep. 2013;30(2):851‐855. doi:10.3892/or.2013.2524 23754096

[152] Ehrenfeld J , Klein U . The key role of the H+ V‐ATPase in acid‐base balance and Na+ transport processes in frog skin. J Exp Biol. 1997;200(Pt 2):247‐256. doi:10.1242/jeb.200.2.247 9050232

[153] Lu Y , Zhang R , Liu S , Zhao Y , Gao J , Zhu L . ZT‐25, a new vacuolar H(+)‐ATPase inhibitor, induces apoptosis and protective autophagy through ROS generation in HepG2 cells. Eur J Pharmacol. 2016;15(771):130‐138. doi:10.1016/j.ejphar.2015.12.026 26689625

[154] Brisson L , Reshkin SJ , Goré J , Roger S . pH regulators in invadosomal functioning: proton delivery for matrix tasting. Eur J Cell Biol. 2012;91(11–12):847‐860. doi:10.1016/j.ejcb.2012.04.004 22673002

[155] Kulshrestha A , Katara GK , Ibrahim SA , et al. In vivo anti‐V‐ATPase antibody treatment delays ovarian tumor growth by increasing antitumor immune responses. Mol Oncol. 2020;14(10):2436‐2454. doi:10.1002/1878-0261.12782 32797726 PMC7530789

[156] Battistone MA , Merkulova M , Park YJ , et al. Unravelling purinergic regulation in the epididymis: activation of V‐ATPase‐dependent acidification by luminal ATP and adenosine. J Physiol. 2019 Apr;597(7):1957‐1973. doi:10.1113/JP277565 30746715 PMC6441927

[157] Fontecha‐Barriuso M , Martín‐Sanchez D , Martinez‐Moreno JM , et al. Molecular pathways driving omeprazole nephrotoxicity. Redox Biol. 2020;32:101464. doi:10.1016/j.redox.2020.101464 32092686 PMC7038587

[158] Mashimo M , Onishi M , Uno A , et al. The 89‐kDa PARP1 cleavage fragment serves as a cytoplasmic PAR carrier to induce AIF‐mediated apoptosis. J Biol Chem. 2021;296:100046. doi:10.1074/jbc.RA120.014479 33168626 PMC7948984

[159] Qi C , Lei L , Hu J , Wang G , Liu J , Ou S . Identification of a five‐gene signature deriving from the vacuolar ATPase (V‐ATPase) sub‐classifies gliomas and decides prognoses and immune microenvironment alterations. Cell Cycle. 2022;10:1‐22. doi:10.1080/15384101.2022.2049157 PMC9132400 35266851

[160] Shiratori R , Furuichi K , Yamaguchi M , et al. Glycolytic suppression dramatically changes the intracellular metabolic profile of multiple cancer cell lines in a mitochondrial metabolism‐dependent manner. Sci Rep. 2019;9(1):18699. doi:10.1038/s41598-019-55296-3 31822748 PMC6904735

[161] Watson MJ , Delgoffe GM . Fighting in a wasteland: deleterious metabolites and antitumor immunity. J Clin Invest. 2022;132(2):e148549. doi:10.1172/JCI148549 35040434 PMC8759785

[162] Dkhil MA , Al‐Quraishy S , Al‐Khalifa MS . The effect of Babesia divergens infection on the spleen of Mongolian gerbils. Biomed Res Int. 2014;2014:483854. doi:10.1155/2014/483854 25136591 PMC4124840

[163] Fedotova EI , Dolgacheva LP , Abramov AY , Berezhnov AV . Lactate and pyruvate activate autophagy and mitophagy that protect cells in toxic model of Parkinson's disease. Mol Neurobiol. 2022;59(1):177‐190. doi:10.1007/s12035-021-02583-8 34642892

[164] Kardideh B , Samimi Z , Norooznezhad F , Kiani S , Mansouri K . Autophagy, cancer and angiogenesis: where is the link? Cell Biosci. 2019;13(9):65. doi:10.1186/s13578-019-0327-6 PMC6693242 31428311

[165] Ding Y , Zhu W , Sun R , et al. Diphenylene iodonium interferes with cell cycle progression and induces apoptosis by modulating NAD(P)H oxidase/ROS/cell cycle regulatory pathways in Burkitt's lymphoma cells. Oncol Rep. 2015;33(3):1434‐1442. doi:10.3892/or.2015.3726 25591797

[166] Zheng J , Dai Y , Lin X , et al. Hypoxia‐induced lactate dehydrogenase a protects cells from apoptosis in endometriosis. Mol Med Rep. 2021;24(3):637. doi:10.3892/mmr.2021.12276 34278456 PMC8281285

[167] Jackson MA , Goodrich JK , Maxan ME , et al. Proton pump inhibitors alter the composition of the gut microbiota. Gut. 2016;65(5):749‐756. doi:10.1136/gutjnl-2015-310861 26719299 PMC4853574

[168] Liu K , Hua S , Song L . PM2.5 exposure and asthma development: the key role of oxidative stress. Oxid Med Cell Longev. 2022;2022:3618806. doi:10.1155/2022/3618806 35419163 PMC9001082

[169] Mendes S , Sá R , Magalhães M , Marques F , Sousa M , Silva E . The role of ROS as a double‐edged sword in (in)fertility: the impact of cancer treatment. Cancers (Basel). 2022;14(6):1585. doi:10.3390/cancers14061585 35326736 PMC8946252

[170] Bai J , Xie N , Hou Y , et al. The enhanced mitochondrial dysfunction by cantleyoside confines inflammatory response and promotes apoptosis of human HFLS‐RA cell line via AMPK/Sirt 1/NF‐κB pathway activation. Biomed Pharmacother. 2022;149:112847. doi:10.1016/j.biopha.2022.112847 35364376

[171] Tang X , Steinman AD , Xue Q , Xu Y , Xie L . Simultaneous electrochemical removal of Microcystis aeruginosa and sulfamethoxazole and its ecologic impacts on Vallisneria spiralis. Sci Total Environ. 2022;1(815):152769. doi:10.1016/j.scitotenv.2021.152769 34990666

[172] Zhang B , Pan C , Feng C , et al. Role of mitochondrial reactive oxygen species in homeostasis regulation. Redox Rep. 2022;27(1):45‐52. doi:10.1080/13510002.2022.2046423 35213291 PMC8890532

[173] Li Y , Yang L , Hou Y , et al. Polydopamine‐mediated graphene oxide and nanohydroxyapatite‐incorporated conductive scaffold with an immunomodulatory ability accelerates periodontal bone regeneration in diabetes. Bioact Mater. 2022;22(18):213‐227. doi:10.1016/j.bioactmat.2022.03.021 PMC8961429 35387166

[174] Han CY , Pichon TJ , Wang X , et al. Leukocyte activation primes fibrinogen for proteolysis by mitochondrial oxidative stress. Redox Biol. 2022 May;51:102263. doi:10.1016/j.redox.2022.102263 35158163 PMC8844908

[175] Xi X , Wang J , Qin Y , You Y , Huang W , Zhan J . The biphasic effect of flavonoids on oxidative stress and cell proliferation in breast cancer cells. Antioxidants (Basel). 2022;11(4):622. doi:10.3390/antiox11040622 35453307 PMC9032920

[176] Zhuo M , Ma J , Quan X . Cytotoxicity of functionalized CeO_2_ nanoparticles towards Escherichia coli and adaptive response of membrane properties. Chemosphere. 2021;281:130865. doi:10.1016/j.chemosphere.2021.130865 34015654

[177] Park JH , Troxel AB , Harvey RG , Penning TM . Polycyclic aromatic hydrocarbon (PAH) o‐quinones produced by the aldo‐keto‐reductases (AKRs) generate abasic sites, oxidized pyrimidines, and 8‐oxo‐dGuo via reactive oxygen species. Chem Res Toxicol. 2006;19(5):719‐728. doi:10.1021/tx0600245 16696575 PMC2366214

[178] Kalegari P , Leme DM , Disner GR , et al. High melanin content in melanoma cells contributes to enhanced DNA damage after rose Bengal photosensitization. Photochem Photobiol. 2022;33(2)192‐196. doi:10.1111/php.13632 35398885

[179] Khurana H , Hazari PP , Mishra AK . Radioprotective efficacy of GSH based peptidomimetic complex of manganese against radiation induced damage: DT(GS)_2_Mn(II). Free Radic Biol Med. 2019;145:161‐174. doi:10.1016/j.freeradbiomed.2019.09.023 31550530

[180] Luciani A , Villella VR , Vasaturo A , et al. Lysosomal accumulation of gliadin p31‐43 peptide induces oxidative stress and tissue transglutaminase‐mediated PPARgamma downregulation in intestinal epithelial cells and coeliac mucosa. Gut. 2010;59(3):311‐319. doi:10.1136/gut.2009.183608 19951908

[181] Коbylinska LI , Boiko NM , Panchuk RR , et al. Putative anticancer potential of novel 4‐thiazolidinone derivatives: cytotoxicity toward rat C6 glioma in vitro and correlation of general toxicity with the balance of free radical oxidation in rats. Croat Med J. 2016;57(2):151‐163. doi:10.3325/cmj.2016.57.151 27106357 PMC4856196

[182] Chueca E , Apostolova N , Esplugues JV , García‐González MA , Lanas Á , Piazuelo E . Proton pump inhibitors display antitumor effects in Barrett's adenocarcinoma cells. Front Pharmacol. 2016;25(7):452. doi:10.3389/fphar.2016.00452 PMC5122752 27932981

[183] Boj‐Carceller D . Proton pump inhibitors: impact on glucose metabolism. Endocrine. 2013;43(1):22‐32. doi:10.1007/s12020-012-9755-3 22886351

[184] Nakagawa S , Arai Y , Kishida T , et al. Lansoprazole inhibits nitric oxide and prostaglandin E(2) production in murine macrophage RAW 264.7 cells. Inflammation. 2012;35(3):1062‐1068. doi:10.1007/s10753-011-9412-7 22134422

[185] Wang Y , Floor E . Hydrogen peroxide inhibits the vacuolar H+‐ATPase in brain synaptic vesicles at micromolar concentrations. J Neurochem. 1998 Feb;70(2):646‐652. doi:10.1046/j.1471-4159.1998.70020646.x 9453558

[186] Gu S , Zhou C , Pei J , et al. Esomeprazole inhibits hypoxia/endothelial dysfunction‐induced autophagy in preeclampsia. Cell Tissue Res. 2022;388(1):181‐194. doi:10.1007/s00441-022-03587-z 35091806 PMC8976802

[187] Griguer CE , Cantor AB , Fathallah‐Shaykh HM , et al. Prognostic relevance of cytochrome C oxidase in primary glioblastoma multiforme. PLoS One. 2013;8(4):e61035. doi:10.1371/journal.pone.0061035 23593382 PMC3622606

[188] Eldridge RC , Uppal K , Hayes DN , et al. Plasma metabolic phenotypes of HPV‐associated versus smoking‐associated head and neck cancer and patient survival. Cancer Epidemiol Biomarkers Prev. 2021;30(10):1858‐1866. doi:10.1158/1055-9965.EPI-21-0576 34376485 PMC8492502

[189] Ieraci L , Eberg M , Forster K , et al. Development of population‐level colon cancer pathway concordance measures and association with survival. Int J Cancer. 2022;150(12):2046‐2057. doi:10.1002/ijc.33964 35170750 PMC9311776

[190] Wang QW , Bao ZS , Jiang T , Zhu YJ . Tumor microenvironment is associated with clinical and genetic properties of diffuse gliomas and predicts overall survival. Cancer Immunol Immunother. 2022;71(4):953‐966. doi:10.1007/s00262-021-03058-4 34535804 PMC10991563

[191] Han Y , Zou C , Zhu C , et al. The systematic landscape of nectin family and nectin‐like molecules: functions and prognostic value in low grade glioma. Front Genet. 2021;1(12):718717. doi:10.3389/fgene.2021.718717 PMC8672115 34925438

[192] Yoneda M , Aklima J , Ohsawa I , Ohta Y . Effects of proton pumping on the structural rigidity of cristae in mitochondria. Arch Biochem Biophys. 2022;15(720):109172. doi:10.1016/j.abb.2022.109172 35276212

[193] Flores‐Romero H , Hohorst L , John M , et al. BCL‐2‐family protein tBID can act as a BAX‐like effector of apoptosis. EMBO J. 2022;41(2):e108690. doi:10.15252/embj.2021108690 34931711 PMC8762556

[194] Huang S , Chen M , Ding X , Zhang X , Zou X . Proton pump inhibitor selectively suppresses proliferation and restores the chemosensitivity of gastric cancer cells by inhibiting STAT3 signaling pathway. Int Immunopharmacol. 2013;17(3):585‐592. doi:10.1016/j.intimp.2013.07.021 23973653

[195] Xie L , Guo YL , Chen YR , et al. A potential drug combination of omeprazole and patchouli alcohol significantly normalizes oxidative stress and inflammatory responses against gastric ulcer in ethanol‐induced rat model. Int Immunopharmacol. 2020;85:106660. doi:10.1016/j.intimp.2020.106660 32559721

[196] Zhao X , Shi X , Liu Q , Li X . Tea polyphenols alleviates acetochlor‐induced apoptosis and necroptosis via ROS/MAPK/NF‐κB signaling in Ctenopharyngodon idellus kidney cells. Aquat Toxicol. 2022;246:106153. doi:10.1016/j.aquatox.2022.106153 35381412

[197] López‐Posadas R , Ballester I , Mascaraque C , et al. Flavonoids exert distinct modulatory actions on cyclooxygenase 2 and NF‐kappaB in an intestinal epithelial cell line (IEC18). Br J Pharmacol. 2010;160(7):1714‐1726. doi:10.1111/j.1476-5381.2010.00827.x 20649574 PMC2936843

[198] Kciuk M , Gielecińska A , Budzinska A , Mojzych M , Kontek R . Metastasis and MAPK pathways. Int J Mol Sci. 2022;23(7):3847. doi:10.3390/ijms23073847 35409206 PMC8998814

[199] Kong L , Barber T , Aldinger J , et al. ROS generation is involved in titanium dioxide nanoparticle‐induced AP‐1 activation through p38 MAPK and ERK pathways in JB6 cells. Environ Toxicol. 2022;37(2):237‐244. doi:10.1002/tox.23393 34730869 PMC9947743

[200] Lommen J , Sus M , Berr K , et al. Analysis of spontaneous and induced osteogenic differentiation in 3D‐micromasses of human multipotent stem cells. In Vivo. 2022 May‐Jun;36(3):1067‐1076. doi:10.21873/invivo.12804 35478128 PMC9087081

[201] Rao Z , Jordan PM , Wang Y , et al. Differential role of vacuolar (H^+^)‐ATPase in the expression and activity of cyclooxygenase‐2 in human monocytes. Biochem Pharmacol. 2020;175:113858. doi:10.1016/j.bcp.2020.113858 32061774

[202] Tian J , Tang C , Wang X , Zhang X , Xiao L , Li W . Supramolecular structure features and immunomodulatory effects of exopolysaccharide from Paecilomyces cicadae TJJ1213 in RAW264.7 cells through NF‐κB/MAPK signaling pathways. Int J Biol Macromol. 2022;15(207):464‐474. doi:10.1016/j.ijbiomac.2022.03.029 35278511

[203] Kanai M , Mullen C , Podolsky DK . Intestinal trefoil factor induces inactivation of extracellular signal‐regulated protein kinase in intestinal epithelial cells. Proc Natl Acad Sci U S A. 1998;95(1):178‐182. doi:10.1073/pnas.95.1.178 9419349 PMC18167

[204] Lee SH , Na SI , Heo JS , et al. Arachidonic acid release by H2O2 mediated proliferation of mouse embryonic stem cells: involvement of Ca2+/PKC and MAPKs‐induced EGFR transactivation. J Cell Biochem. 2009;106(5):787‐797. doi:10.1002/jcb.22013 19199341

[205] Choi S , Yeum CH , Kim YD , et al. Receptor tyrosine and MAP kinase are involved in effects of H(2)O(2) on interstitial cells of Cajal in murine intestine. J Cell Mol Med. 2010;14(1–2):257‐266. doi:10.1111/j.1582-4934.2008.00403.x 20414970 PMC3837618

[206] Margalef P , Colomer C , Villanueva A , et al. BRAF‐induced tumorigenesis is IKKα‐dependent but NF‐κB‐independent. Sci Signal. 2015;8(373):ra38. doi:10.1126/scisignal.2005886 25900832

[207] Mazzio EA , Soliman KFA . Whole‐transcriptomic profile of SK‐MEL‐3 melanoma cells treated with the histone deacetylase inhibitor: trichostatin A. Cancer Genomics Proteomics. 2018;15(5):349‐364. doi:10.21873/cgp.20094 30194076 PMC6199573

[208] Kessler T , Sahm F , Sadik A , et al. Molecular differences in IDH wildtype glioblastoma according to MGMT promoter methylation. Neuro Oncol. 2018;20(3):367‐379. doi:10.1093/neuonc/nox160 29016808 PMC5817966

[209] Huang J , Yu J , Tu L , Huang N , Li H , Luo Y . Isocitrate dehydrogenase mutations in glioma: from basic discovery to therapeutics development. Front Oncol. 2019;12(9):506. doi:10.3389/fonc.2019.00506 PMC6584818 31263678

[210] Terrasi A , Bertolini I , Martelli C , et al. Specific V‐ATPase expression sub‐classifies IDHwt lower‐grade gliomas and impacts glioma growth in vivo. EBioMedicine. 2019;41:214‐224. doi:10.1016/j.ebiom.2019.01.052 30737087 PMC6441867

[211] Wronska E , Polkowski M , Orlowska J , Mroz A , Wieszczy P , Regula J . Argon plasma coagulation for Barrett's esophagus with low‐grade dysplasia: a randomized trial with long‐term follow‐up on the impact of power setting and proton pump inhibitor dose. Endoscopy. 2021;53(2):123‐132. doi:10.1055/a-1203-5930 32650347

[212] Lombardi G , Rumiato E , Bertorelle R , et al. Clinical and genetic factors associated with severe hematological toxicity in glioblastoma patients during radiation plus Temozolomide treatment: a prospective study. Am J Clin Oncol. 2015;38(5):514‐519. doi:10.1097/COC.0b013e3182a790ea 24064758

[213] Camelo‐Piragua S , Kesari S . Further understanding of the pathology of glioma: implications for the clinic. Expert Rev Neurother. 2016;16(9):1055‐1065. doi:10.1080/14737175.2016.1194755 27228211

[214] Crettol S , Petrovic N , Murray M . Pharmacogenetics of phase I and phase II drug metabolism. Curr Pharm des. 2010;16(2):204‐219. doi:10.2174/138161210790112674 19835560

[215] Prasad H , Rao R . Histone deacetylase‐mediated regulation of endolysosomal pH. J Biol Chem. 2018;293(18):6721‐6735. doi:10.1074/jbc.RA118.002025 29567836 PMC5936830

[216] Scheuch E , Walter R , Hadasová E , Amon I , Siegmund W . Influence of H2‐receptor‐ and proton pump inhibitors on some functions of the oxydative and conjugative drug metabolism. Pharmazie. 1996;51(7):493‐497. 8774841

[217] Piotrowski AF , Blakeley J . Clinical Management of Seizures in patients with low‐grade glioma. Semin Radiat Oncol. 2015;25(3):219‐224. doi:10.1016/j.semradonc.2015.02.009 26050593 PMC4696025

[218] Moliterno JA , Patel TR , Piepmeier JM . Neurosurgical approach. Cancer J. 2012;18(1):20‐25. doi:10.1097/PPO.0b013e3183243f6e3 22290253

[219] Huberfeld G , Vecht CJ . Seizures and gliomas—towards a single therapeutic approach. Nat Rev Neurol. 2016;12(4):204‐216. doi:10.1038/nrneurol.2016.26 26965673

[220] Zhu K , Sun J , Kang Z , et al. Repurposing of omeprazole for oligodendrocyte differentiation and remyelination. Brain Res. 2019;1(1710):33‐42. doi:10.1016/j.brainres.2018.12.037 30590025

[221] Yenisehirli A , Onur R . Positive inotropic and negative chronotropic effects of proton pump inhibitors in isolated rat atrium. Eur J Pharmacol. 2005;519(3):259‐266. doi:10.1016/j.ejphar.2005.06.040 16125697

[222] Chen YY , Tsai CF , Tsai MC , Hsu YW , Lu FJ . Inhibitory effects of rosmarinic acid on pterygium epithelial cells through redox imbalance and induction of extrinsic and intrinsic apoptosis. Exp Eye Res. 2017;160:96‐105. doi:10.1016/j.exer.2017.05.008 28559202

[223] Sun W , Zhang W , Yu J , Lu Z , Yu J . Inhibition of Nrf2 might enhance the anti‐tumor effect of temozolomide in glioma cells via inhibition of Ras/Raf/MEK signaling pathway. Int J Neurosci. 2021;131(10):975‐983. doi:10.1080/00207454.2020.1766458 32378973

[224] Kim MJ , Jeon JH . Recent advances in understanding Nrf2 Agonism and its potential clinical application to metabolic and inflammatory diseases. Int J Mol Sci. 2022;23(5):2846. doi:10.3390/ijms23052846 35269986 PMC8910922

[225] Li N , Zhan X . Machine learning identifies Pan‐cancer landscape of Nrf2 oxidative stress response pathway‐related genes. Oxid Med Cell Longev. 2022;17(2022):8450087. doi:10.1155/2022/8450087 PMC8886747 35242279

[226] Zhou J , Li XY , Liu YJ , et al. Full‐coverage regulations of autophagy by ROS: from induction to maturation. Autophagy. 2021;18:1‐16. doi:10.1080/15548627.2021.1984656 34662529 PMC9225210

[227] Ji XJ , Chen SH , Zhu L , et al. Knockdown of NF‐E2‐related factor 2 inhibits the proliferation and growth of U251MG human glioma cells in a mouse xenograft model. Oncol Rep. 2013;30(1):157‐164. doi:10.3892/or.2013.2476 23673813

[228] Fan J , Lv H , Li J , et al. Roles of Nrf2/HO‐1 and HIF‐1α/VEGF in lung tissue injury and repair following cerebral ischemia/reperfusion injury. J Cell Physiol. 2019;234(6):7695‐7707. doi:10.1002/jcp.27767 30565676

[229] Du R , Lu KV , Petritsch C , et al. HIF1alpha induces the recruitment of bone marrow‐derived vascular modulatory cells to regulate tumor angiogenesis and invasion. Cancer Cell. 2008;13(3):206‐220. doi:10.1016/j.ccr.2008.01.034 18328425 PMC2643426

[230] Yang Y , Wang X , Zhang J , et al. Abnormal phenotype of Nrf2 is associated with poor prognosis through hypoxic/VEGF‐A‐Rap1b/VEGFR2 pathway in gastric cancer. Aging (Albany NY). 2022;14(7):3293‐3312. doi:10.18632/aging.204013 35417854 PMC9037254

[231] Zhao J , Niu X , Yu J , et al. Poria cocos polysaccharides attenuated ox‐LDL‐induced inflammation and oxidative stress via ERK activated Nrf2/HO‐1 signaling pathway and inhibited foam cell formation in VSMCs. Int Immunopharmacol. 2020;80:106173. doi:10.1016/j.intimp.2019.106173 31945610

[232] Foresti R , Bains SK , Pitchumony TS , et al. Small molecule activators of the Nrf2‐HO‐1 antioxidant axis modulate heme metabolism and inflammation in BV2 microglia cells. Pharmacol Res. 2013;76:132‐148. doi:10.1016/j.phrs.2013.07.010 23942037

[233] Seelige R , Washington A Jr , Bui JD . The ancient cytokine IL‐17D is regulated by Nrf2 and mediates tumor and virus surveillance. Cytokine. 2017;91:10‐12. doi:10.1016/j.cyto.2016.11.017 27940089 PMC5316352

[234] Huang M , Mehrabi Nasab E , Athari SS . Immunoregulatory effect of mesenchymal stem cell via mitochondria signaling pathways in allergic asthma. Saudi J Biol Sci. 2021 Dec;28(12):6957‐6962. doi:10.1016/j.sjbs.2021.07.071 34866995 PMC8626264

[235] Grauer O , Pöschl P , Lohmeier A , Adema GJ , Bogdahn U . Toll‐like receptor triggered dendritic cell maturation and IL‐12 secretion are necessary to overcome T‐cell inhibition by glioma‐associated TGF‐beta2. J Neurooncol. 2007;82(2):151‐161. doi:10.1007/s11060-006-9274-2 17106649

[236] Li J , Zhao L , Zhang Y , et al. Imbalanced immune responses involving inflammatory molecules and immune‐related pathways in the lung of acute and subchronic arsenic‐exposed mice. Environ Res. 2017;159:381‐393. doi:10.1016/j.envres.2017.08.036 28843991

[237] Lee S , Hur EG , Ryoo IG , Jung KA , Kwak J , Kwak MK . Involvement of the Nrf2‐proteasome pathway in the endoplasmic reticulum stress response in pancreatic β‐cells. Toxicol Appl Pharmacol. 2012;264(3):431‐438. doi:10.1016/j.taap.2012.08.021 22959925

[238] Garufi A , Pistritto G , D'Orazi V , Cirone M , D'Orazi G . The impact of NRF2 inhibition on drug‐induced colon cancer cell death and p53 activity: a pilot study. Biomolecules. 2022;12(3):461. doi:10.3390/biom12030461 35327653 PMC8946796

[239] Catanzaro E , Calcabrini C , Turrini E , Sestili P , Fimognari C . Nrf2: a potential therapeutic target for naturally occurring anticancer drugs? Expert Opin Ther Targets. 2017;21(8):781‐793. doi:10.1080/14728222.2017.1351549 28675319

[240] Zhang HF , Wang JH , Wang YL , et al. Salvianolic acid a protects the kidney against oxidative stress by activating the Akt/GSK‐3*β*/Nrf2 signaling pathway and inhibiting the NF‐*κ*B signaling pathway in 5/6 Nephrectomized rats. Oxid Med Cell Longev. 2019;18(2019):2853534. doi:10.1155/2019/2853534 PMC6442489 31011401

[241] Gan X , Zhang R , Gu J , et al. Acidic microenvironment regulates the severity of hepatic ischemia/reperfusion injury by modulating the generation and function of Tregs via the PI3K‐mTOR pathway. Front Immunol. 2020;9(10):2945. doi:10.3389/fimmu.2019.02945 PMC6962105 31998287

[242] Gravano DM , Vignali DA . The battle against immunopathology: infectious tolerance mediated by regulatory T cells. Cell Mol Life Sci. 2012;69(12):1997‐2008. doi:10.1007/s00018-011-0907-z 22205213 PMC3353028

[243] Rolim GB , Dantas Lima AJP , Dos Santos Cardoso VI , et al. Can inflammasomes promote the pathophysiology of glioblastoma multiforme? A view about the potential of the anti‐inflammasome therapy as pharmacological target. Crit Rev Oncol Hematol. 2022;172:103641. doi:10.1016/j.critrevonc.2022.103641 35189327

[244] Zhang Y , Feng Z , Liu J , et al. Polarization of tumor‐associated macrophages by TLR7/8 conjugated radiosensitive peptide hydrogel for overcoming tumor radioresistance. Bioact Mater. 2022;3(16):359‐371. doi:10.1016/j.bioactmat.2021.12.033 PMC8965723 35386314

[245] Wedekind H , Walz K , Buchbender M , et al. Head and neck tumor cells treated with hypofractionated irradiation die via apoptosis and are better taken up by M1‐like macrophages. Strahlenther Onkol. 2022;198(2):171‐182. doi:10.1007/s00066-021-01856-4 34665291 PMC8789708

[246] Katara GK , Kulshrestha A , Jaiswal MK , Pamarthy S , Gilman‐Sachs A , Beaman KD . Inhibition of vacuolar ATPase subunit in tumor cells delays tumor growth by decreasing the essential macrophage population in the tumor microenvironment. Oncogene. 2016;35(8):1058‐1065. doi:10.1038/onc.2015.159 25961933

[247] Davuluri GVN , Chan CH . Regulation of intrinsic and extrinsic metabolic pathways in tumor‐associated macrophages. FEBS J. 2022;105(33):111‐113. doi:10.1111/febs.16465 PMC10711806 35486022

[248] Zhang W , Shi Y , Li H , et al. In situ injectable nano‐complexed hydrogel based on chitosan/dextran for combining tumor therapy via hypoxia alleviation and TAMs polarity regulation. Carbohydr Polym. 2022;15(288):119418. doi:10.1016/j.carbpol.2022.119418 35450661

[249] Vishvakarma NK , Singh SM . Augmentation of myelopoiesis in a murine host bearing a T cell lymphoma following in vivo administration of proton pump inhibitor pantoprazole. Biochimie. 2011;93(10):1786‐1796. doi:10.1016/j.biochi.2011.06.022 21722701

[250] Vishvakarma NK , Singh SM . Immunopotentiating effect of proton pump inhibitor pantoprazole in a lymphoma‐bearing murine host: implication in antitumor activation of tumor‐associated macrophages. Immunol Lett. 2010;134(1):83‐92. doi:10.1016/j.imlet.2010.09.002 20837061

[251] Jindal A , Bruzzì S , Sutti S , et al. Fat‐laden macrophages modulate lobular inflammation in nonalcoholic steatohepatitis (NASH). Exp Mol Pathol. 2015;99(1):155‐162. doi:10.1016/j.yexmp.2015.06.015 26112094

[252] Rentscher KE , Carroll JE , Polsky LR , Lamkin DM . Chronic stress increases transcriptomic indicators of biological aging in mouse bone marrow leukocytes. Brain Behav Immun Health. 2022;12(22):100461. doi:10.1016/j.bbih.2022.100461 PMC9035650 35481228

[253] Lee HJ , Park JM , Han YM , et al. The role of chronic inflammation in the development of gastrointestinal cancers: reviewing cancer prevention with natural anti‐inflammatory intervention. Expert Rev Gastroenterol Hepatol. 2016;10(1):129‐139. doi:10.1586/17474124.2016.1103179 26524133

[254] Kotsuka M , Hashimoto Y , Nakatake R , et al. Omeprazole increases survival through the inhibition of inflammatory mediaters in two rat sepsis models. Shock. 2022;57(3):444‐456. doi:10.1097/SHK.0000000000001897 34923545 PMC8868211

[255] Gao H , Hoesel LM , Guo RF , Rancilio NJ , Sarma JV , Ward PA . Adenoviral‐mediated overexpression of SOCS3 enhances IgG immune complex‐induced acute lung injury. J Immunol. 2006;177(1):612‐620. doi:10.4049/jimmunol.177.1.612 16785559

[256] Ishibashi K , Koguchi T , Matsuoka K , et al. Interleukin‐6 induces drug resistance in renal cell carcinoma. Fukushima J Med Sci. 2018;64(3):103‐110. doi:10.5387/fms.2018-15 30369518 PMC6305783

[257] Sehgal A . Molecular changes during the genesis of human gliomas. Semin Surg Oncol. 1998;14(1):3‐12. doi:10.1002/(sici)1098-2388(199801/02)14:1<3::aid-ssu2>3.0.co;2-f 9407626

[258] Rupert JE , Narasimhan A , Jengelley DHA , et al. Tumor‐derived IL‐6 and trans‐signaling among tumor, fat, and muscle mediate pancreatic cancer cachexia. J Exp Med. 2021;218(6):e20190450. doi:10.1084/jem.20190450 33851955 PMC8185651

[259] Rydén M , Arvidsson E , Blomqvist L , Perbeck L , Dicker A , Arner P . Targets for TNF‐alpha‐induced lipolysis in human adipocytes. Biochem Biophys Res Commun. 2004;318(1):168‐175. doi:10.1016/j.bbrc.2004.04.010 15110769

[260] Li J , Chen XL , Shaker A , et al. Contribution of immunomodulators to gastroesophageal reflux disease and its complications: stromal cells, interleukin 4, and adiponectin. Ann N Y Acad Sci. 2016;1380(1):183‐194. doi:10.1111/nyas.13157 27441783 PMC5083128

[261] Thomas L , Rao Z , Gerstmeier J , et al. Selective upregulation of TNFα expression in classically‐activated human monocyte‐derived macrophages (M1) through pharmacological interference with V‐ATPase. Biochem Pharmacol. 2017;15(130):71‐82. doi:10.1016/j.bcp.2017.02.004 28189727

[262] Kedika RR , Souza RF , Spechler SJ . Potential anti‐inflammatory effects of proton pump inhibitors: a review and discussion of the clinical implications. Dig Dis Sci. 2009;54(11):2312‐2317. doi:10.1007/s10620-009-0951-9. 19714466 PMC3035917

[263] Kim YJ , Lee JS , Hong KS , Chung JW , Kim JH , Hahm KB . Novel application of proton pump inhibitor for the prevention of colitis‐induced colorectal carcinogenesis beyond acid suppression. Cancer Prev Res (Phila). 2010;3(8):963‐974. doi:10.1158/1940-6207.CAPR-10-0033 20628001

[264] Lee H , Koh JY . Roles for H+ /K+‐ATPase and zinc transporter 3 in cAMP‐mediated lysosomal acidification in bafilomycin A1‐treated astrocytes. Glia. 2021;69(5):1110‐1125. doi:10.1002/glia.23952 33314298

[265] Ye WF , Liu J , Wan L , et al. Effect of Xinfeng capsule on AS patients and their serum immunoglobulin subtypes and peripheral lymphocyte autophagy. Zhongguo Zhong Xi Yi Jie He Za Zhi. 2016;36(3):310‐316. 27236888

[266] Li T , Han J , Jia L , Hu X , Chen L , Wang Y . PKM2 coordinates glycolysis with mitochondrial fusion and oxidative phosphorylation. Protein Cell. 2019;10(8):583‐594. doi:10.1007/s13238-019-0618-z 30887444 PMC6626593

[267] Wang Y , Han X , Fu M , et al. Qiliqiangxin attenuates hypoxia‐induced injury in primary rat cardiac microvascular endothelial cells via promoting HIF‐1α‐dependent glycolysis. J Cell Mol Med. 2018;22(5):2791‐2803. doi:10.1111/jcmm.13572 29502357 PMC5908112

[268] Chen Z , Dong H , Jia C , et al. Activation of mTORC1 in collecting ducts causes hyperkalemia. J Am Soc Nephrol. 2014;25(3):534‐545. doi:10.1681/ASN.2013030225 24203997 PMC3935580

[269] Li J , Zhang T , Ren T , et al. Oxygen‐sensitive methylation of ULK1 is required for hypoxia‐induced autophagy. Nat Commun. 2022;13(1):1172. doi:10.1038/s41467-022-28831-6 35246531 PMC8897422

[270] Basit F , van Oppen LM , Schöckel L , et al. Mitochondrial complex I inhibition triggers a mitophagy‐dependent ROS increase leading to necroptosis and ferroptosis in melanoma cells. Cell Death Dis. 2017;8(3):e2716. doi:10.1038/cddis.2017.133 28358377 PMC5386536

[271] Sanfelice RADS , Bortoleti BTDS , Tomiotto‐Pellissier F , et al. Biogenic silver nanoparticles (AgNp‐bio) reduce toxoplasma gondii infection and proliferation in HeLa cells, and induce autophagy and death of tachyzoites by apoptosis‐like mechanism. Acta Trop. 2021;222:106070. doi:10.1016/j.actatropica.2021.106070 34331897

[272] Lu ZN , Shi ZY , Dang YF , et al. Pantoprazole pretreatment elevates sensitivity to vincristine in drug‐resistant oral epidermoid carcinoma in vitro and in vivo. Biomed Pharmacother. 2019 Dec;120:109478. doi:10.1016/j.biopha.2019.109478 31568987

[273] Wang H , Wang A , Wang X , Zeng X , Xing H . AMPK/PPAR‐γ/NF‐κB axis participates in ROS‐mediated apoptosis and autophagy caused by cadmium in pig liver. Environ Pollut. 2022;294:118659. doi:10.1016/j.envpol.2021.118659 34896222

[274] Yue X , Wen S , Long‐Kun D , et al. Three important short‐chain fatty acids (SCFAs) attenuate the inflammatory response induced by 5‐FU and maintain the integrity of intestinal mucosal tight junction. BMC Immunol. 2022;23(1):19. doi:10.1186/s12865-022-00495-3 35448938 PMC9027456

[275] Bosnjak T , Solberg R , Hemati PD , Jafari A , Kassem M , Johansen HT . Lansoprazole inhibits the cysteine protease legumain by binding to the active site. Basic Clin Pharmacol Toxicol. 2019;125(2):89‐99. doi:10.1111/bcpt.13230 30916878

[276] Ebrahimpour A , Wang M , Li L , et al. Esomeprazole attenuates inflammatory and fibrotic response in lung cells through the MAPK/Nrf2/HO1 pathway. J Inflamm (Lond). 2021;18(1):17. doi:10.1186/s12950-021-00284-6 34011367 PMC8136131

[277] Schnöller LE , Albrecht V , Brix N , et al. Integrative analysis of therapy resistance and transcriptomic profiling data in glioblastoma cells identifies sensitization vulnerabilities for combined modality radiochemotherapy. Radiat Oncol. 2022;17(1):79. doi:10.1186/s13014-022-02052-z 35440003 PMC9020080

[278] Ma C , Nguyen HPT , Jones JJ , et al. Extracellular vesicles secreted by glioma stem cells are involved in radiation resistance and glioma progression. Int J Mol Sci. 2022;23(5):2770. doi:10.3390/ijms23052770 35269915 PMC8911495

[279] Yu Y , Wang A , Wang S , et al. Efficacy of temozolomide‐conjugated gold nanoparticle photothermal therapy of drug‐resistant glioblastoma and its mechanism study. Mol Pharm. 2022;19(4):1219‐1229. doi:10.1021/acs.molpharmaceut.2c00083 35262365

[280] Ortiz R , Perazzoli G , Cabeza L , et al. Temozolomide: an updated overview of resistance mechanisms, nanotechnology advances and clinical applications. Curr Neuropharmacol. 2021;19(4):513‐537. doi:10.2174/1570159X18666200626204005 32589560 PMC8206461

[281] Averbeck D , Rodriguez‐Lafrasse C . Role of mitochondria in radiation responses: epigenetic, metabolic, and signaling impacts. Int J Mol Sci. 2021;22(20):11047. doi:10.3390/ijms222011047 34681703 PMC8541263

[282] Tomita K , Kuwahara Y , Igarashi K , et al. Mitochondrial dysfunction in diseases, longevity, and treatment resistance: tuning mitochondria function as a therapeutic strategy. Genes (Basel). 2021;12(9):1348. doi:10.3390/genes12091348 34573330 PMC8467098

[283] Tričković JF , Šobot AV , Joksić I , Joksić G . Telomere fragility in radiology workers occupationally exposed to low doses of ionising radiation. Arh Hig Rada Toksikol. 2022;73(1):23‐30. doi:10.2478/aiht-2022-73-3609 35390241 PMC8999593

[284] Lo Greco MC , Milazzotto R , Liardo RLE , et al. Relapsing high‐grade glioma from peritumoral zone: critical review of radiotherapy treatment options. Brain Sci. 2022;12(4):416. doi:10.3390/brainsci12040416 35447948 PMC9027370

[285] Hebert KA , Jaramillo S , Yu W , et al. Esomeprazole enhances the effect of ionizing radiation to improve tumor control. Oncotarget. 2021;12(14):1339‐1353. doi:10.18632/oncotarget.28008 34262645 PMC8274720

[286] Cao L , Zhu Y , Wang W , Wang G , Zhang S , Cheng H . Emerging nano‐based strategies against drug resistance in tumor chemotherapy. Front Bioeng Biotechnol. 2021;7(9):798882. doi:10.3389/fbioe.2021.798882 PMC8688801 34950650

[287] Zhang Q , Ding J , Wang Y , He L , Xue F . Tumor microenvironment manipulates chemoresistance in ovarian cancer (review). Oncol Rep. 2022;47(5):102. doi:10.3892/or.2022.8313 35362546

[288] Hsu HH , Chen MC , Baskaran R , et al. Oxaliplatin resistance in colorectal cancer cells is mediated via activation of ABCG2 to alleviate ER stress induced apoptosis. J Cell Physiol. 2018;233(7):5458‐5467. doi:10.1002/jcp.26406 29247488

[289] Xue YY , Lu YY , Sun GQ , et al. CN‐3 increases TMZ sensitivity and induces ROS‐dependent apoptosis and autophagy in TMZ‐resistance glioblastoma. J Biochem Mol Toxicol. 2022;36(3):e22973. doi:10.1002/jbt.22973 34967073

[290] Lustig SD , Kodali SK , Longo SL , Kundu S , Viapiano MS . Ko143 reverses MDR in glioblastoma *via* deactivating P‐glycoprotein, sensitizing a resistant phenotype to TMZ treatment. Anticancer Res. 2022;42(2):723‐730. doi:10.21873/anticanres.15530 35093870

[291] Liu M , Tang R , Jiang Y . Pantoprazole induces apoptosis of leukemic cells by inhibiting expression of P‐glycoprotein/multidrug resistance‐associated Protein‐1 through PI3K/AKT/mTOR signaling. Indian J Hematol Blood Transfus. 2017;33(4):500‐508. doi:10.1007/s12288-017-0808-x 29075060 PMC5640553

[292] Bianchi G , Ravera S , Traverso C , et al. Curcumin induces a fatal energetic impairment in tumor cells in vitro and in vivo by inhibiting ATP‐synthase activity. Carcinogenesis. 2018;39(9):1141‐1150. doi:10.1093/carcin/bgy076 29860383

[293] Kang R , Tang D , Schapiro NE , et al. The HMGB1/RAGE inflammatory pathway promotes pancreatic tumor growth by regulating mitochondrial bioenergetics. Oncogene. 2014;33(5):567‐577. doi:10.1038/onc.2012.631 23318458 PMC3795800

[294] Rodón J , Carducci M , Sepulveda‐Sánchez JM , et al. Pharmacokinetic, pharmacodynamic and biomarker evaluation of transforming growth factor‐β receptor I kinase inhibitor, galunisertib, in phase 1 study in patients with advanced cancer. Invest New Drugs. 2015;33(2):357‐370. doi:10.1007/s10637-014-0192-4 25529192 PMC4387272

[295] Peppicelli S , Bianchini F , Toti A , Laurenzana A , Fibbi G , Calorini L . Extracellular acidity strengthens mesenchymal stem cells to promote melanoma progression. Cell Cycle. 2015;14(19):3088‐3100. doi:10.1080/15384101.2015.1078032 26496168 PMC4825622

[296] Gao H , Wang X , Qu X , et al. Omeprazole attenuates cisplatin‐induced kidney injury through suppression of the TLR4/NF‐κB/NLRP3 signaling pathway. Toxicology. 2020;440:152487. doi:10.1016/j.tox.2020.152487 32418911

[297] Babu D , Mudiraj A , Yadav N , Y B V K C , Panigrahi M , Prakash Babu P . Rabeprazole has efficacy per se and reduces resistance to temozolomide in glioma via EMT inhibition. Cell Oncol (Dordr). 2021 Aug;44(4):889‐905. doi:10.1007/s13402-021-00609-w 33948872 PMC12980772

[298] Hada N , Netzer WJ , Belhassan F , Wennogle LP , Gizurarson S . Nose‐to‐brain transport of imatinib mesylate: a pharmacokinetic evaluation. Eur J Pharm Sci. 2017;1(102):46‐54. doi:10.1016/j.ejps.2017.02.032 28238945

[299] Kulkarni P , Haldar MK , Katti P , et al. Hypoxia responsive, tumor penetrating lipid nanoparticles for delivery of chemotherapeutics to pancreatic cancer cell spheroids. Bioconjug Chem. 2016;27(8):1830‐1838. doi:10.1021/acs.bioconjchem.6b00241 27391789 PMC5502747

[300] Matsumoto S , Kishimoto S , Saito K , et al. Metabolic and physiologic imaging biomarkers of the tumor microenvironment predict treatment outcome with radiation or a hypoxia‐activated prodrug in mice. Cancer Res. 2018;78(14):3783‐3792. doi:10.1158/0008-5472.CAN-18-0491 29792309 PMC8092078

[301] Ouar Z , Bens M , Vignes C , et al. V andewalle a. inhibitors of vacuolar H+‐a TPase impair the preferential accumulation of daunomycin in lysosomes and reverse the resistance to anthracyclines in drug‐resistant renal epithelial cells. Biochem J. 2003;370:185‐193. doi:10.1042/BJ20021411 12435274 PMC1223162

[302] De Milito A , Fais S . Tumor acidity, chemoresistance and proton pump inhibitors. Future Oncol. 2005;1:779‐786. doi:10.2217/14796694.1.6.779 16556057

[303] Menon A , Abraham AG , Mahfouz M , et al. Concomitant use of proton pump inhibitors with Capecitabine based Neoadjuvant Chemoradiotherapy for locally advanced rectal cancer: is it Safe? Am J Clin Oncol. 2021;44(9):487‐494. doi:10.1097/COC.0000000000000850 34269694

[304] Zhang JL , Liu M , Yang Q , et al. Effects of omeprazole in improving concurrent chemoradiotherapy efficacy in rectal cancer. World J Gastroenterol. 2017;23(14):2575‐2584. doi:10.3748/wjg.v23.i14.2575 28465642 PMC5394521

[305] Eguchi K , Suzuki M , Ida S , et al. Successful treatment of radiation‐induced mucositis with proton pump inhibitor administration: a report of two laryngeal cancer cases. Auris Nasus Larynx. 2017;44(1):122‐125. doi:10.1016/j.anl.2016.05.006 27264874

[306] Su Q , Wang D , Yuan B , Liu F , Lei Y , Li S . Effects of proton pump inhibitors on lung cancer precise radiotherapy‐induced radiation pneumonitis. Cell Biochem Biophys. 2014;70(2):1113‐1117. doi:10.1007/s12013-014-0030-5 24858285 PMC4182645

[307] Xun X , Yin Q , Fu Y , He X , Dong Z . Proton pump inhibitors and the risk of community‐acquired pneumonia: an updated meta‐analysis. Ann Pharmacother. 2022;56(5):524‐532. doi:10.1177/10600280211039240 34425689

[308] Naghibzadeh N , Salmani F , Nomiri S , Tavakoli T . Investigating the effect of quadruple therapy with saccharomyces boulardii or lactobacillus reuteri strain (DSMZ 17648) supplements on eradication of helicobacter pylori and treatments adverse effects: a double‐blind placebo‐controlled randomized clinical trial. BMC Gastroenterol. 2022;22(1):107. doi:10.1186/s12876-022-02187-z 35255819 PMC8903632

[309] Bataille P , Chasset F , Monfort JB , et al. Cutaneous drug‐induced lupus erythematosus: clinical and immunological characteristics and update on new associated drugs. Ann Dermatol Venereol. 2021;133:56‐62. 10.1016/j.annder.2021.02.006 34711400

[310] Kamal F , Khan MA , Molnar MZ , Howden CW . The association between proton pump inhibitor use with acute kidney injury and chronic kidney disease. J Clin Gastroenterol. 2018;52(6):468‐476. doi:10.1097/MCG.0000000000001035 29668562

[311] Rameau A , Andreadis K , Bayoumi A , Kaufman M , Belafsky P . Side effects of proton pump inhibitors: what are Patients' concerns? J Voice. 2021;35(5):809.e15‐809.e20. doi:10.1016/j.jvoice.2020.01.018 32093923

[312] Jakubowski JK , Patel R , Buddharaju V . Polymyositis presenting as rhabdomyolysis after the initiation of omeprazole. Cureus. 2020;12(5):e8125. doi:10.7759/cureus.8125 32432010 PMC7234030

[313] Gamaletsou MN , Denning DW . Gastroesophageal reflux disease and pulmonary diseases associated with Aspergillosis: is there a connection? Mycopathologia. 2017;182(11–12):1125‐1129. doi:10.1007/s11046-017-0176-y 28702854

[314] Jimenez L , Campos Codo A , Sampaio VS , et al. Acid pH increases SARS‐CoV‐2 infection and the risk of death by COVID‐19. Front Med (Lausanne). 2021;8:637885. doi:10.3389/fmed.2021.637885 34490283 PMC8417536

[315] Vinke P , Wesselink E , van Orten‐Luiten W , van Norren K . The use of proton pump inhibitors may increase symptoms of muscle function loss in patients with chronic illnesses. Int J Mol Sci. 2020;21(1):323. doi:10.3390/ijms21010323 31947724 PMC6981685

[316] Shikh EV , Makhova AA , Chemeris AV , Tormyshov IA . Iatrogenic deficits of micronutrients. Vopr Pitan. 2021;90(4):53‐63. Russian. doi:10.33029/0042-8833-2021-90-4-53-63 34538035

[317] Dries LS , Haefliger R , Seibert BS , de Lima AG , Cardoso CO , Perassolo MS . Cognition, oxidative stress and vitamin B12 levels evaluation on patients under long‐term omeprazole use. J Pharm Pharmacol. 2022;74(4):547‐555. doi:10.1093/jpp/rgab001 33793790

[318] Liew JW , Peloquin C , Tedeschi SK , et al. Proton pump inhibitors and risk of calcium pyrophosphate deposition in a population‐based study. Arthritis Care Res (Hoboken). 2022;35(12):371‐378. doi:10.1002/acr.24876 PMC9440954 35245410

[319] Douwes RM , Vinke JSJ , Gomes‐Neto AW , et al. Type of proton‐pump inhibitor and risk of iron deficiency in kidney transplant recipients ‐ results from the TransplantLines biobank and cohort study. Transpl Int. 2021;34(11):2305‐2316. doi:10.1111/tri.14110 34519109 PMC9293430

[320] Vanwing V , Schevenels S , Klockaerts C , Danckaerts M . Psychotische symptomen als bijwerking van omeprazol [psychotic symptoms as a side‐effect of omeprazole]. Tijdschr Psychiatr. 2018;60(12):834‐837. 30536296

